# A prophage-encoded actin-like protein required for efficient viral DNA replication in bacteria

**DOI:** 10.1093/nar/gkv374

**Published:** 2015-04-27

**Authors:** Catriona Donovan, Antonia Heyer, Eugen Pfeifer, Tino Polen, Anja Wittmann, Reinhard Krämer, Julia Frunzke, Marc Bramkamp

**Affiliations:** 1 Department of Biology I, Ludwig-Maximilians-University Munich, Großhaderner Str. 2–4, 82152 Planegg-Martinsried, Germany; 2 Institute for Biochemistry, University of Cologne, Zülpicherstr. 47, 50674 Cologne, Germany; 3 Institut für Bio- und Geowissenschaften, IBG-1: Biotechnologie, Forschungszentrum Jülich, D-52425 Jülich, Germany

## Abstract

In host cells, viral replication is localized at specific subcellular sites. Viruses that infect eukaryotic and prokaryotic cells often use host-derived cytoskeletal structures, such as the actin skeleton, for intracellular positioning. Here, we describe that a prophage, CGP3, integrated into the genome of *Corynebacterium glutamicum* encodes an actin-like protein, AlpC. Biochemical characterization confirms that AlpC is a *bona fide* actin-like protein and cell biological analysis shows that AlpC forms filamentous structures upon prophage induction. The co-transcribed adaptor protein, AlpA, binds to a consensus sequence in the upstream promoter region of the *alpAC* operon and also interacts with AlpC, thus connecting circular phage DNA to the actin-like filaments. Transcriptome analysis revealed that *alpA* and *alpC* are among the early induced genes upon excision of the CGP3 prophage. Furthermore, qPCR analysis of mutant strains revealed that both AlpA and AlpC are required for efficient phage replication. Altogether, these data emphasize that AlpAC are crucial for the spatio-temporal organization of efficient viral replication. This is remarkably similar to actin-assisted membrane localization of eukaryotic viruses that use the actin cytoskeleton to concentrate virus particles at the egress sites and provides a link of evolutionary conserved interactions between intracellular virus transport and actin.

## INTRODUCTION

DNA of viral origin, including fully functional prophages, cryptic (degenerated) prophage elements or phage morons, represents a common element of bacterial genomes (1,2). Upon prophage induction, e.g. triggered by the host SOS response, temperate phages enter the lytic pathway leading to the excision of the viral DNA from the genome, replication, virion assembly and lysis of the host cell (3,4). Recent reports revealed that viral replication in prokaryotes appears to be organized at specific intracellular locations and this process relies on the action of cytoskeletal proteins (5).

Cytoskeletal elements in eu- and prokaryotes are involved in a variety of different cellular processes (6,7). During mitotic segregation, eukaryotic chromosomes are moved by microtubules that attach to the centromere (6,8). In bacterial cells, mechanistically similar DNA segregation processes have been described (7,9–16). Best understood is the segregation of plasmid DNA via a tripartite *par*titioning system. The genetic organization of *par* loci is similar for both chromosome- and plasmid-encoded systems. In general, the *par* locus entails two *trans*-acting proteins encoded in an operon and *cis*-acting ‘centromere-like’ elements. The centromere-binding protein binds the centromere-like element forming a nucleoprotein complex (17–19). The segrosomes are recognized and segregated by the action of a partition protein, which, depending on the plasmid partitioning system, is either a Walker-A P-loop ATPase (ParA, Type I), an actin-like ATPase (ParM, Type II) or a tubulin-like GTPase (TubZ, Type III) (14,15,20–22). The precise partitioning mechanism remains under debate (23). Dynamic cytomotive filament pushing or pulling mechanisms have been supported for most actin and tubulin based segregation systems (21,24,25). Walker-A P-loop ATPase, on the other hand, have been proposed to employ a diffusion-ratchet model. In this model, the nucleotide bound from of the ATPase determines its affinity for the nucleoid. Combined with associated regulatory elements and intrinsic ATPase activity, gradients of the Walker-A ATPases form on the nucleoid. Rounds of nucleoid tethering and releasing lead to a directed movement of the DNA cargo (26,27). Active partitioning systems are imperative for efficient maintenance of low copy plasmids, such as P1 prophage from *Escherichia coli* (28–30).

Actin-like proteins also play a major role in cell growth and shape determination. MreB is the archetype of the bacterial cytoskeletal proteins (31–33). The MreB structure revealed homology to actin (34). MreB and its homologs are now known to be involved in the positioning of cell wall synthesizing complexes (35–38). Remarkably, MreB was also shown to be involved in viral replication. Replication of the *Bacillus subtilis* phage *φ*29 depends on the presence of all three MreB isoforms (MreB, Mbl and MreBH) (39–41). MreB apparently interacts with a phage-encoded membrane protein p16.7. Similarly, replication of the *E. coli* phage PRD1 is reduced upon inhibition of MreB. Recently, a tubulin-like protein, PhuZ, from bacteriophage 201φ2-1 was described to form spindle-like filaments *in vivo* thereby positioning the phage DNA within the cell (42,43).

In this study, we identified and characterized a novel actin-like protein encoded by the cryptic prophage CGP3 located in the genome of *Corynebacterium glutamicum* strain ATCC 13032. The genome of this important industrial platform organism harbors three cryptic prophages, CGP1–3, of which only CGP3 has been shown to replicate extra-chromosomally in a circularized form (44). CGP3 encompasses with 187 kb almost 6% of the entire *C. glutamicum* genome and belongs to the largest phage elements with known sequence (45). A cluster of tRNA genes is found on the left periphery of the CGP3 phage, while the right border encodes a putative phage integrase. The element is flanked by conserved attachment sites (44). Spontaneous induction of the CGP3 phage in a subpopulation of cells (1–3%) has been reported previously (44). However, CGP3 appeared to be inactive in terms of cell lysis and virion production and is therefore referred to as a cryptic prophage, which likely became trapped in the genome in the course of evolution.

Here, we describe that the first open reading frame in the CGP3 prophage encodes an actin-like protein, AlpC, and adjacent a phage DNA-binding protein, AlpA. Both AlpC and AlpA are necessary for efficient phage replication *in vivo*. Further, we show that AlpA binds a consensus sequence on the phage DNA molecules. The actin-like protein AlpC assembles into filaments that interact with the AlpA bound CGP3 DNA, which may function as a scaffold for the organization efficient viral replication. *Corynebacterium glutamicum* does not encode an MreB homlog, thus it would seem advantageous that the *C. glutamicum* CGP3 prophage encodes its own cytoskeletal element. Our data suggest that bacterial phages use an actin-based transport system, analogous to vertebrate viruses such as the herpesvirus which use host cell derived cytoskeletal elements (46,47).

## MATERIALS AND METHODS

### Recombinant DNA work

Standard methods like PCR, restriction or ligation were carried out according to established protocols (48,49). Oligonucleotide synthesis and DNA sequencing was performed by Eurofins MWG Operon (Ebersfeld, Germany). Strains, plasmids and oligonucleotides are listed in Supplementary Table S1. Strain construction is described in the Supplemental Materials and Methods.

### Determination of circular phage DNA using quantitative PCR

The relative amount of circular phage DNA was determined *via* quantitative PCR (qPCR). Therefore, *C. glutamicum* wild type, the *alpC* deletion strain, and the *alpA* deletion strain were grown in 5 ml BHI (Brain Heart Infusion, Difco) for about 6 h at 30°C. A second precultivation was performed in CGXII minimal medium containing 4% glucose as carbon source. From each preculture two main cultures were inoculated to an OD_600_ of 1 in CGXII minimal medium. At an OD_600_ of 3 mitomycin C (final concentration of 0.6 μM) was added to one culture to induce the SOS response; the second, untreated culture served as reference. Five milliliters samples were harvested by centrifugation (4000 x *g*, 10 min and 4°C) at different time points after induction (0–24 h). The preparation of genomic DNA was performed as described previously (50).

The determination of the relative amount of circular DNA of the phage CGP3 by quantitative PCR was conducted according to the protocol described in Frunzke *et al*. (44). Briefly, each sample contained 1 μg total DNA as a template for amplification. Amplification was performed using the DyNAmo Capillary SYBR Green qPCR Kit (Finnzymes Oy, Vantaa) and a LightCycler type 1.0 (Roche Diagnostics). The *ddh* gene (cg2900), which is present in one copy in the *C. glutamicum* genome, served as reference gene for normalization; the oligonucleotides ddh-LC-for and ddh-LC-rev were used for amplification. For the detection of circular CGP3 DNA, oligonucleotides (Phage-LC-for and Phage-LC-rev) were designed which anneal to the left and right boarder of CGP3, pointing into divergent directions. This arrangement was used to specifically amplify a 150 bp DNA fragment covering the attachment site (*attP*) of the excised circular CGP3 DNA molecule. For the calculation of the relative amount of the circular phage DNA, the amount of the circular phage DNA in induced cells was normalized with the amount of circular phage DNA in uninduced cells of the same strain at each time point.

### DNA microarrays

For transcriptome analysis cells were cultivated as described for the determination of circular CGP3 DNA (previous paragraph). Transcriptomes of *C. glutamicum* ATCC 13032 were comparisons of cells treated with or without 0.6 mM mitomycin C after 1, 3, 6 and 9 h after addition of mitomycin C. The custom DNA microarrays were obtained from Agilent Technologies (Waldbronn, Germany). Agilent's eArray platform was used to design oligonucleotide probes with the best probe methodology and assemble custom 4×44K 60mer microarray designs (https://earray.chem.agilent.com/earray/). The custom design included oligonucleotides for the annotated protein-coding genes and structural RNA genes of the four bacterial genomes from *C. glutamicum, Escherichia coli, Gluconobacter oxydans* and *B. subtilis* for genome-wide gene expression analysis. For *C. glutamicum*, the genome annotation NC_006958 from NCBI was used listing 3057 protein coding genes and 80 structural tRNA and ribosomal RNA genes (45). In the custom design, *C. glutamicum* genes are represented by one, two or three oligonucleotides which were used to determine relative RNA levels. The custom array design also included the Agilent's control spots. Purified cDNA samples to be compared were pooled and prepared two-color samples were hybridized on 4×44K arrays at 65°C for 17 h using Agilent's gene expression hybridization kit, Agilent's hybridization chamber and Agilent's hybridization oven. After hybridization the arrays were washed using Agilent's wash buffer kit according to the manufacturer's instructions. Fluorescence of hybridized DNA microarrays was determined at 532 nm (Cy3-dUTP) and 635 nm (Cy5-dUTP) at 5 μm resolution with a GenePix 4000B laser scanner and GenePix Pro 6.0 software (Molecular Devices, Sunnyvale, CA, USA). Fluorescence images were saved to raw data files in TIFF format (GenePix Pro 6.0). Quantitative TIFF image analysis was carried out using GenePix image analysis software and the Agilent's gene array list (GAL) file. The results were saved as GPR-file (GenePix Pro 6.0). For background correction of spot intensities, ratio calculation and ratio normalization, GPR-files were processed using the BioConductor R-packages limma and marray (http://www.bioconductor.org). For further analysis, the processed and lowess-normalized data as well as detailed experimental information according to MIAME (51) were stored in the in house DNA microarray database (52). To search the data for differentially expressed genes by the processed Cy5/Cy3 ratio reflecting the relative RNA level, the criteria flags ≥0 (GenePix Pro 6.0) and signal/noise ≥5 for Cy5 (F635Median/B635Median) or Cy3 (F532Median/B532Median) were used. Array data were deposited in the GEO database (ncbi.nlm.nih.gov/geo) under accession number GSE45907.

### Heterologous protein expression and purification

His_10_-AlpC was heterogolously overproduced in *E. coli* BL21 (DE3) pLysS. Expression was induced with 0.4 mM isopropyl-β-d-thiogalactopyranoside (IPTG). Cells were collected by centrifugation at 5000 × *g* (4°C) for 10 min, resuspended in buffer A (100 mM Tris/HCl, pH 7.5, 150 mM KCl, 150 mM NaCl, 10% glycerol and 10 mM imidazole), supplemented with DNase I and protease inhibitor. The cleared cell lysate was applied to a 1 ml HisTrap™ FF column, washed with 10 column volumes of buffer A and subsequently eluted by a step gradient of buffer B (buffer A supplemented with 490 mM imidazole). The affinity purified AlpC protein was further applied to a Superdex^TM^ 200 10/300 gel filtration column and eluted in buffer C (buffer A lacking imidazole). Eluted fractions were analyzed by sodium dodecyl sulphate-polyacrylamide gel electrophoresis (SDS-PAGE) and immunoblotting. Heterologous overexpression and purification of AlpC^D301A^ was identical.

The production of His_10_-AlpA in *E. coli* BL21 (DE3) pLysS was induced with 0.5 mM IPTG and harvested after 4 h of expression as described for His_10_-AlpC. The disruption of the cells and purification of the protein with a Ni^2+^-NTA column (nickel-nitriloacetic acid) (Qiagen, Hilden) were performed as described previously (53). The elution of AlpA was conducted with TNI400 buffer (20 mM Tris/HCl, 300 mM NaCl, 400 mM imidazole). The fractions of eluted protein were pooled and the buffer was exchanged to binding buffer (20 mM Tris/HCl, pH 7.5, 50 mM KCl, 10 mM MgCl_2_, 5% (v/v) glycerol, 0.5 mM ethylenediaminetetraacetic acid (EDTA), 0.005% (w/v) Triton X-100) with a PD10 desalting column (GE Healthcare) for DNA–protein binding studies.

### Electrophoretic mobility shift assays

Studies of the DNA–protein binding of AlpA and potential target DNA were performed according to (54). The DNA (500 bp) of the upstream region of *alpA* and downstream region of *alpC* were amplified by PCR. The promoter region of cg2036, a gene encoded by the prophage CGP3 in *C. glutamicum*, was tested as control fragment. 100 ng DNA per lane were incubated with different molar ratios of purified AlpA (0–500-fold molar excess) for 20 min before loading to a nondenaturing 10% polyacrylamide gel.

### Nucleotide hydrolysis assay

ATPase and GTPase activity was measured in a coupled enzyme assay constantly regenerating ATP/GTP, allowing monitoring of ATP hydrolysis over time (55). The regeneration of ATP/GTP is coupled to the oxidation of nicotinamide adenine dinucleotide phophate (NADH). When ADP/GDP is converted back to ATP/GTP pyruvate kinase converts phosphoenolpyruvate (PEP) to pyruvate. Lactate dehydrogenase then converts the pyruvate to lactate, resulting in the oxidation of one NADH molecule. The decrease of NADH absorbance at 340 nm is monitored over time.

Nucleotide hydrolysis rate of 1 μM protein was performed in buffer containing 50 mM Tris/HCl pH 7.5, 150 mM NaCl, 10% glycerol. The reaction was performed in a total volume of 100 μl containing varying nucleotide concentration, equimolar MgSO_4_, 1 mM PEP, 0.6 mM NADH, 20 U/ml pyruvate kinase and 20 U/ml lactate dehydrogenase. The samples were set-up in a 96-well microtiter plate and the absorbance of NADH was monitored at 340 nm for 1 h at 30°C (Tecan Plate Reader, software: I-control). All data were obtained from triplicate determination and corrected for nucleotide autohydrolysis.

### Sedimentation assay

Prior to experimental setup, protein samples were subjected to centrifugation at 120 000 × *g* for 10 min to remove any aggregated protein. Purified protein (2 μM) was mixed with nucleotide (2 μM) in the presence or absence of 2 μM Mg^2+^ or 4 μM EDTA. Volumes were adjusted to 100 μl with buffer C. Reaction mixtures were incubated at 30°C for 30 min. Higher-ordered AlpC protein complexes were then sedimentated by centrifugation at 120 × 000 *g* for 10 min. Supernatant and pellet fractions were separated and quantitatively analyzed by immunoblot.

For quantitative western blot analyses His_10_-AlpC and His_10_-AlpC^D301A^ from the sedimentation assays, samples were blotted onto an Immobilon-FL PVDF membrane (Millipore) and probed with anti-His antibody (Qiagen), followed by IRDye 800-conjugated goat anti-mouse IgG (H + L) antibodies (LI-COR) as secondary antibody. The IR fluorescence signals were quantified with the OdysseyTM IR fluorescence scanning system (LI-COR). Background values were automatically subtracted using the Odyssey software (LICOR) (Median Top/Bottom method). Signals were normalized to a protein only sample.

### Co-sedimentation assay

To test for interaction of AlpA and AlpC, co-sedimentation assays with purified proteins were performed. AlpC and AlpA were mixed in equimolar ratio (5 μM, respectively, in a reaction volume of 20 μl) in EMSA buffer (20 mM Tris/HCl, pH 7.5, 50 mM KCl, 10 mM MgCl_2_, 5% (v/v) glycerol, 0.5 mM EDTA and 0.005% (w/v) Triton X-100). The mixture was incubated at 30°C for 30 min in the presence or absence of 1 mM ATP and/or 100 ng DNA (500 bp fragment of the upstream region of *alpA*). The reaction mixture was centrifuged at 16 100 × *g* for 1 h. The supernatant was withdrawn and the sedimented protein was resuspended in the same volume. Both fractions were separated on a 12% SDS-PAGE and visualized by Coomassie staining. The amount of sedimented AlpA protein was quantified by using ImageQuant TL software (GE Healthcare).

### Fluorescence microscopy

For microscopic examination, expression from the pEKEx2 plasmid was induced by addition of 0.5 mM IPTG to the growth medium, for ∼60–90 min. For co-visualization of eCFP-AlpC filament and induced CGP3 prophage, cells were grown in CGXII medium supplemented with 4% glucose. Excision of the CGP3 prophage was induced by addition of mitomycin C (final concentration 0.6 μg/ml or as indicated in text). Approximately 30 min prior to microscopic examination, 0.1 mM IPTG was added to induce synthesis of YFP-TetR. AlpC-mCherry expressed from its native promoter was induced by addition of 5 μM mitomycin C.

For phase contrast and fluorescence microscopy, 1–3 μl of a culture sample was placed on a microscope slide coated with a thin 1% agarose layer and covered by a cover slip. Images were taken on a Zeiss AxioImager M1 equipped with a Zeiss AxioCam HRm camera or on a Zeiss AxioImager M2 equipped with a Zeiss AxioCam MRm camera. GFP fluorescence was monitored using filter set 38 HE eGFP, BG-430 fluorescence and CFP (eCFP) were monitored using filter 47 HE CFP, red fluorescence (membrane stain) was monitored by using filter 43 HE Cy3 or filter 63He and DAPI / Hoechst fluorescence was examined with filter set 49. An EC Plan-Neofluar 100x/1.3 Oil Ph3 objective was used. Digital images were acquired and analyzed with the AxioVision 4.6 software (Carl Zeiss). Final image preparation was done using Adobe Photoshop 6.0 (Adobe Systems Incorporated).

Time lapse and FRAP analysis was carried out using a Delta Vision Elite (GE Healthcare, Applied Precision) equipped with an Insight SSI™ illumination, an X4 laser module and a CoolSnap HQ2 CCD camera. Images were taken with a 100× oil PSF U-Plan S-Apo 1.4 NA objective.

### FRAP analysis

Fluorescence recovery after photobleaching experiments were performed using a *C. glutamicum* strain expressing AlpC-mCherry under control of the native promoter, but encoded on a plasmid (pEC-XC99E-P*_alpA_*-*alpC*-*mcherry*). Induction of AlpC expression was initiated with 5 μM mitomycin C addition to exponentially growing cells. Cells were mounted on BHI agar pads at 30°C. Cells were imaged using an DeltaVision Elite system (GE Healthcare) using the SSI illumination system with the following settings: mCherry excitation 50% SSI with 0,2 s exposure. Bleaching was done with a 561 nm laser (50 mW) at 10% power and a 100x Oil PSF Objective (U-PLAN S-APO 100X Oil, 1.4NA, 0.12 WD). The duration of the laser pulse was 0.01 s. Time lapse image series were taken every 10 s and the laser event was placed after the first image. Dark-state reversal of the mCherry fluorophore was controlled for by fixing cells with 1% formaldehyde prior to image analysis (30 min room temperature). Images were analyzed with Fiji and values were normalized for both bleaching and cytoplasmic background fluorescence. Relative values were used in order to allow comparison between cells, where a value of 1 corresponds to the fluorescence intensity before the bleaching event and a value of 0 corresponds to the cytoplasmic background fluorescence. The cytoplasmic background fluorescence value is the average between the cytoplasmic fluorescence of three cells selected from the analyzed microscope image. Subtraction of cytoplasmic fluorescence from the original fluorescence value highlights filament dependent fluorescent signals.

\begin{equation*}
\begin{array}{l} {\rm Filament}\;{\rm fluorescence} = {\rm total}\;{\rm fluorescence} - \\
({\rm cytoplasmic}\;{\rm background}\;{\rm fluorescence}\;{\rm average}*{\rm cell}\;{\rm area}) \\
\end{array}
\end{equation*}

Bleaching due to imaging was calculated by averaging the decrease of relative fluorescence in three independent areas of the microscope image not affected by the laser bleaching event. Images were assembled in Adobe Photoshop and graphs plotted with Microsoft Excel.

## RESULTS

### A prophage-encoded cytoskeletal protein

**Figure 1.**
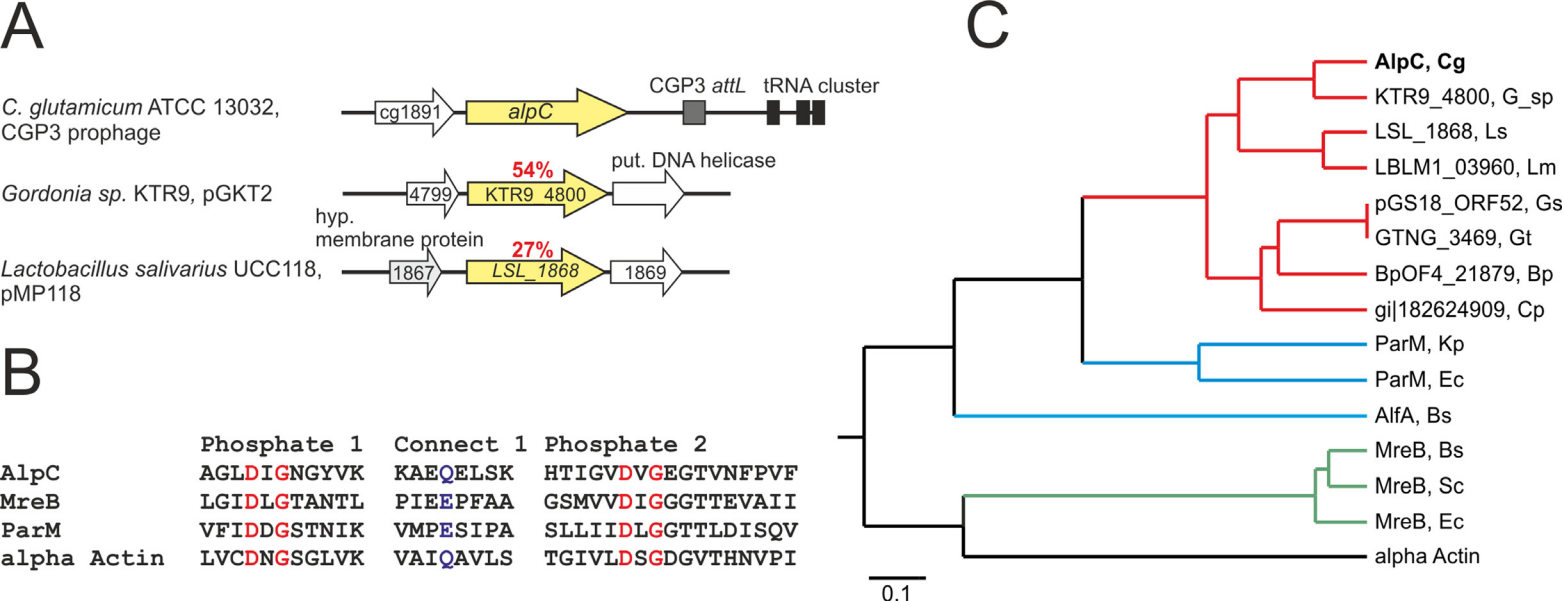
Genetic organization and phylogenetic analysis of AlpC. (**A**) Genetic organization of *alpC* and homologous proteins (yellow). (**B**) Alignment of the phosphate 1 (red), connect 1 (blue) and phosphate 2 (red) regions (57). The residues of *C. glutamicum* ATCC 13032 AlpC, *B. subtilis* MreB, *E. coli* ParM and human alpha actin are shown. Highlighted residues correspond to alpha actin D13 and G15 (red), Q139 (blue), and D156 and G158 (red). (**C**) Phylogenetic tree visualizing the relationship of AlpC homologs to bacterial actin-like proteins and human alpha actin. Branches shown in green indicate MreB proteins, branches colored in blue indicate plasmid partitioning ParM and AlfA proteins. AlpC and homologous proteins (red) are closer related to ParM. Amino acid sequences were aligned using the neighbor-joining method (ClustalW): AlpC Cg, *C. glutamicum* ATCC 13032 (gi|62390556), KTR9_4800 G_sp, Gordonia sp. KTR9 (gi|301321491), LSL_1868 Ls, *Lactobacillus salivarius* UCC118 (gi|90962843), LBLM1_03960 Lm, *Lactobacillus mucosae* LM1 (gi|377831150), pGS18_ORF52 Gs, *Geobacillus stearothermophilus* (gi|169636508) (plasmid-encoded), GTNG_3469 Gt, *Geobacillus thermodenitrificans* NG80-2 (gi|138898362), BpOF4_21879 Bp, *Bacillus pseudofirmus* OF4 (gi|288557196), Cp, *Clostridium perfringens* D str. JGS1721 (gi|182624909), ParM Kp, *Klebsiella pneumonia* (gi|146150982), ParM Ec, *E. coli* (gi|385721336), MreB Bs, *B. subtilis* (gi|255767642), MreB Sc, *Streptomyces coelicolor* (gi|21221069), MreB Ec, *E. coli* (gi|377940072), alpha actin, *Homo sapiens* (gi|178029).

In previous work, our labs have shown that the *C. glutamicum* ATCC 13032 prophage CGP3 is able to excise from the chromosome and replicate autonomously (44). The first open reading frame in the prophage, cg1890, shows low sequence identity to bacterial actins (Figure 1). Recently, a phylogenetic analysis identified Cg1890 as an actin-like protein (56). Cg1890 shares considerably low sequence identity (mostly <30%) with actin and other actin-like proteins, however the typical actin signature motif is conserved (Figure 1B). Hence, we renamed Cg1890 to AlpC (designated AlpC, for Actin-Like Protein *Corynebacterium*). The actin signature motifs are involved in binding nucleotide in the presence of a divalent cation and catalyzing the transfer of a phosphoryl group from ATP to a hydroxyl group (57,58). Amide residues from the conserved loops of the phosphate 1 and phosphate 2 motifs are involved in hydrogen bonding with the β- and γ-phosphates of ATP (58). The connect 1 sequence makes interactions with the metal ion complexed to ATP.

A phylogenetic search revealed that among actin-like proteins AlpC and homologous proteins are closer related to plasmid partitioning systems (ParM) than to MreB-type cytoskeletal proteins (Figure 1C). Homologous proteins of *C. glutamicum* AlpC were found to be, in most cases, plasmid-encoded and were identified in a variety of Gram-positive bacteria such as *Lactobacillus* and *Bacillus* species as well as the pathogen *Clostridium perfringes* (Figure 1C). While the phylogenetic analysis carried out by Derman and coworkers identified of a large number of actin-like proteins (56), the role of phage-encoded actin-like proteins has not been studied in further detail, yet.

### AlpC hydrolyzes nucleotides *in vitro*

**Figure 2.**
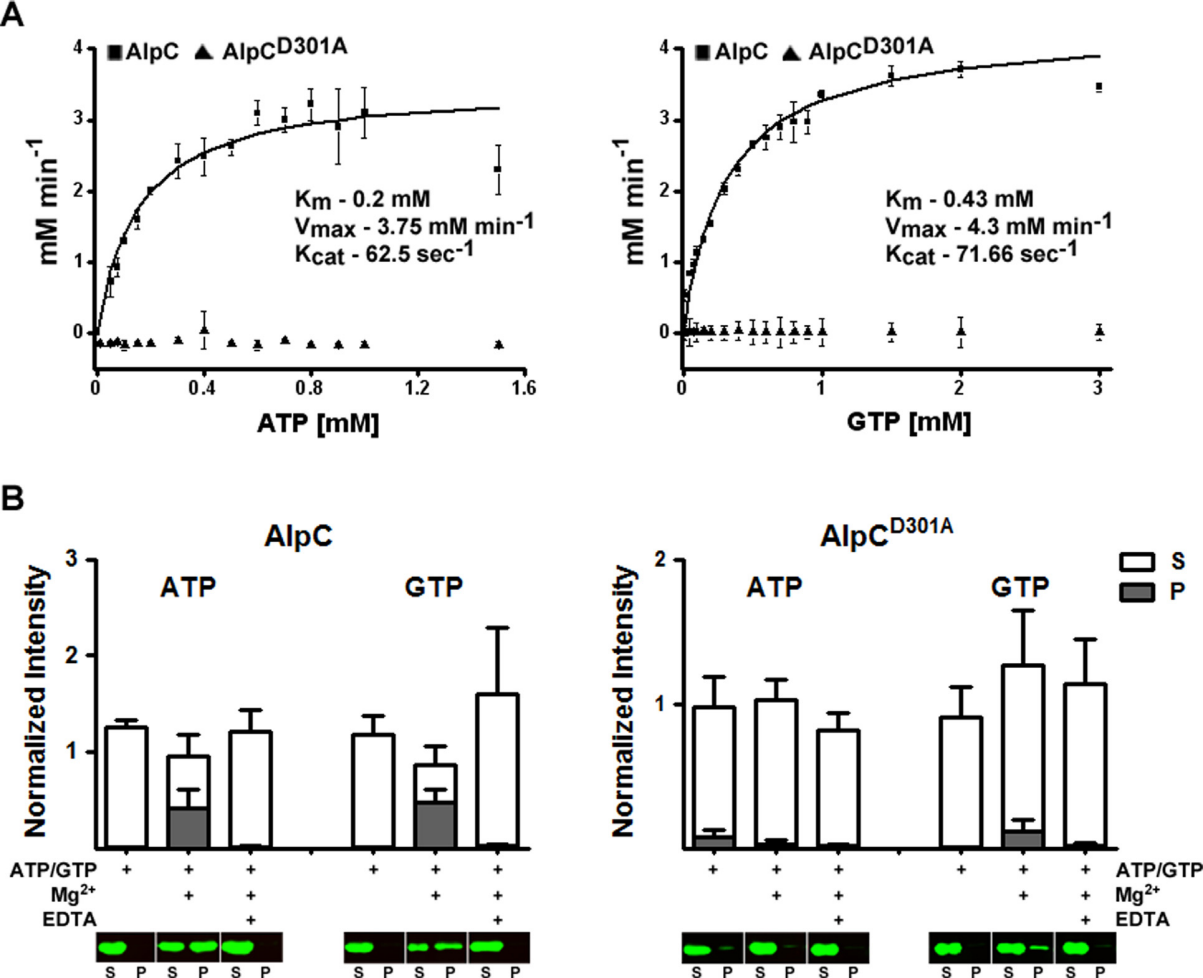
AlpC hydrolyses ATP and GTP, *in vitro*. (**A**) Nucleotide hydrolysis was assayed using a coupled assay in which ATP/GTP was continuously regenerated from ADP/GDP (see Materials and Methods). The nucleotide hydrolysis rate was assayed in the presence of 1 μM protein with increasing concentration of ATP/GTP. Plots are fitted for Michaelis–Menten kinetics. The hydrolysis kinetics of AlpC is similar for both ATP and GTP. Mutation of the conserved aspartic acid of the phosphate 2 motif of AlpC (AlpC^D301A^) abrogates nucleotide hydrolysis. Error bars indicate the standard error of three independent experiments. (**B**) AlpC assembles into higher-ordered protein complexes. Polymerization of 2 μM AlpC or AlpC^D301A^ was assayed at 30°C in the presence of 2 mM nucleotide (ATP or GTP), 2 mM Mg^2+^ and/or 4 mM EDTA, as indicated, and detected by means of sedimentation assays and quantitative Western blot. All fluorescence intensities are normalized against AlpC or AplC^D301A^ protein samples lacking nucleotide, Mg^2+^ or EDTA. Sedimentation of AlpC is dependent on the presence of nucleotide and Mg^2+^. In the presence of EDTA sedimentation of AlpC is abolished. Sedimentation of the hydrolytic inactive mutant (AlpC^D301A^) was observed (in the presence of ATP only or GTP and Mg^2+^), however, significantly reduced compared to the wild-type AlpC protein. In the presence of EDTA AlpC^D301A^ did not sediment. A montage of the immunoblotted samples is shown in the lower part of the figure. S, supernatant; P, pellet. Error bars indicate the standard error of three independent experiments.

A distinguishing feature of actin and actin related proteins is the ability to hydrolyze nucleotides. In order to analyze nucleotide hydrolysis of AlpC, a recombinant His-tagged variant of AlpC and a mutant AlpC variant lacking the conserved aspartic acid of the phosphate 2 motif (AlpC^D301A^) were overproduced in the heterogolous bacterium *E. coli* and purified. In a nucleotide hydrolysis assay, the activity of 1 μM of purified protein was measured with increasing concentrations of ATP or GTP (Figure 2A). Akin to other characterized actin-like proteins, AlpC can hydrolyze both ATP and GTP, and exhibits Michaelis–Menten kinetics. In the presence of ATP, the maximum turnover rate is reached at slightly lower substrate concentrations compared to GTP. In the presence of GTP, AlpC has a *V*_max_ of 4.3 mM min^−1^, compared to 3.75 mM min^−1^ for ATP (Figure 2A). The measured *K*_m_ is low and does not differ significantly between ATP and GTP, 0.2 mM and 0.43 mM, respectively. *In vivo*, the more abundant ATP is probably the favored substrate. Nucleotide hydrolysis is abolished in the AlpC^D301A^ mutant (Figure 2A).

### AlpC assembles into higher-ordered oligomeric complexes, *in vitro* and *in vivo*

One of the defining properties of actin and related cytoskeletal proteins is the ability to polymerize into filamentous structures *in vivo*. To determine if AlpC assembles into higher-ordered oligomeric complexes *in vitro*, sedimentation assays were performed (Figure 2B). After removal of potential protein aggregates by centrifugation, 2 μM of purified protein was incubated for 30 min at 30°C in the presence or absence of nucleotide, Mg^2+^ and EDTA as indicated in Figure 2B. The higher-ordered oligomeric AlpC complexes were isolated by centrifugation. Sedimentation of AlpC was dependent on the presence of nucleotide (ATP or GTP) and Mg^2+^. Addition of the metal chelator EDTA abolished sedimentation of AlpC, ruling out sedimentation of protein aggregates. The inactive mutant AlpC^D301A^ did not exhibit a nucleotide-dependent sedimentation behavior (Figure 2B). Sedimentation of AlpC^D301A^ was observed when incubated in the presence of ATP only or GTP and Mg^2+^, however assembly into higher-ordered AlpC^D301A^ complexes was not observed when incubated in the presence of nucleotide, Mg^2+^ and EDTA

**Figure 3.**
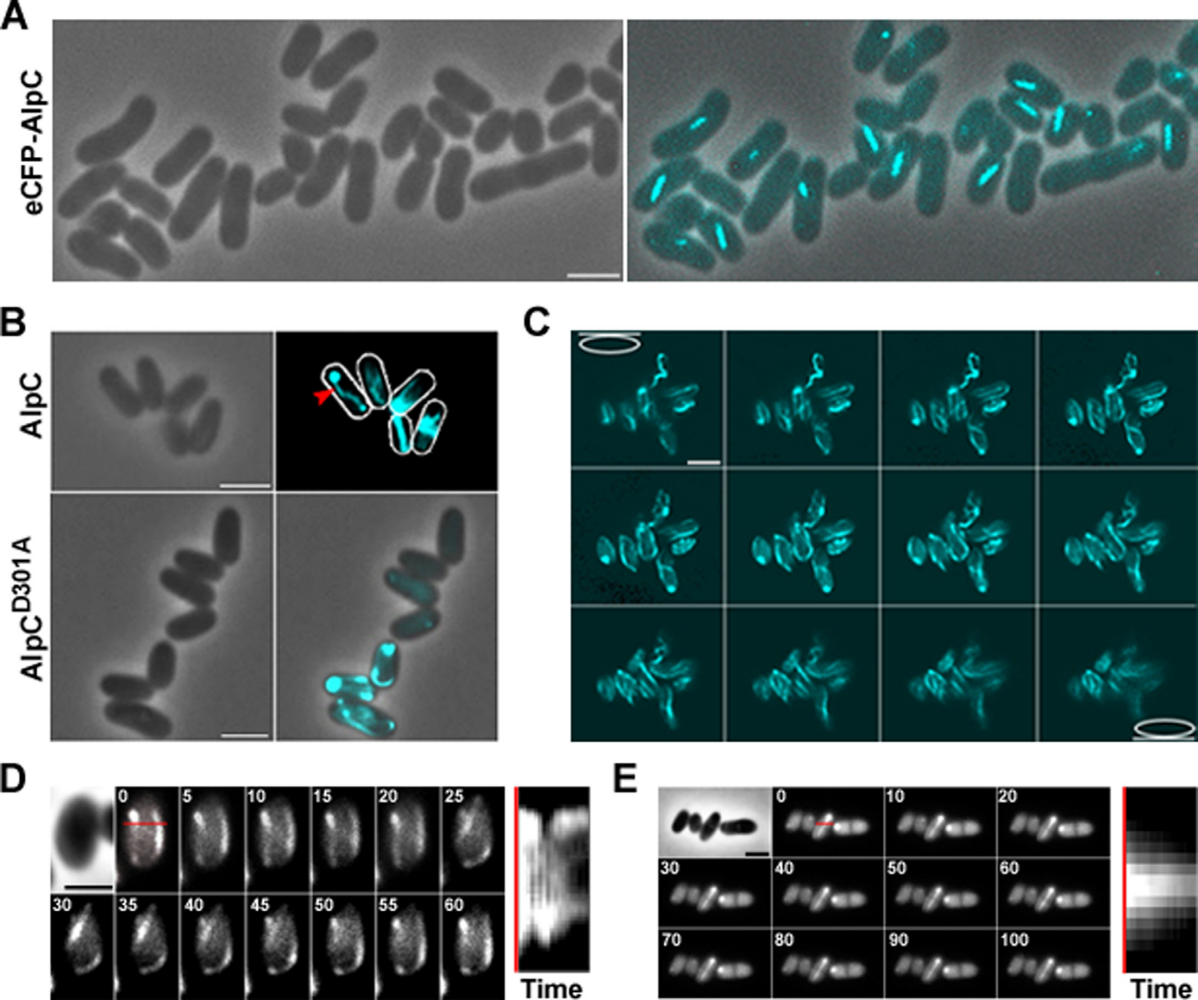
AlpC assembles into filaments in *C. glutamicum*. (**A**) eCFP-AlpC (CDC020) assembles into short, straight filaments when expressed at physiological concentration. Cells were grown in CGXII containing 4% glucose. Excision of the CGP3 prophage was induced with 0.6 μg/ml mitomycin C for 3 h prior to microscopic analysis. (**B**) AlpC-CFP (CDC021) assembles into long, curved filaments when overproduced in *C. glutamicum*, red arrowhead. Cells were grown in BHI and induced by addition of 0.5 mM IPTG 1 h prior to microscopic analysis. Mutation of the conserved aspartic acid of the phosphate 2 motif of AlpC reduces filament assembly (AlpC^D301A^-CFP, CDC022) (lower panel). (**C**) Z-stack analysis of the subcellular distribution of AlpC-CFP (CDC021) filaments. Cells overproducing AlpC-CFP contain numerous filaments of various orientation, curvature and length (Movie S1). (**D**) AlpC-CFP filaments are dynamic. Images were acquired at 5 s intervals (top left) for 1 min (Movie S2). The red line denotes the position in the cell used to generate the kymograph (right). (**E**) AlpC^D301A^-CFP filaments are static. Images were acquired at 5 s intervals (top left) for 100 s (Movie S3). As in (C), a kymograph is shown on the right. Scale bar, 2 μm

In order to study the behavior of AlpC at physiological concentration, the native *alpC* locus was replaced with an *ecfp-alpC* allele (CDC020, Supplementary Table S1). Under conditions of prophage induction using mitomycin C, eCFP-AlpC (CDC020) readily assembled into filaments (Figure 3A). AlpC filaments or foci were observed in the vast majority of cells. The filaments were varying in length, straight and mostly found pointing to the cell membrane at different angles. These results suggest that AlpC expression and filament formation occurs in response to induction of the CGP3 prophage.

The low level of eCFP-AlpC (CDC020) produced when expressed at physiological concentration made it unsuitable for further analysis, such as analysis of filament distribution or time lapse. Consequently, *alpC-cfp*, encoding a fusion protein of AlpC and CFP, was expressed from a pEKEx2 plasmid (CDC021, Supplementary Table S1), bringing the fusion gene under control of an IPTG inducible promoter. In the majority of cells (97.2%, *n* = 870), AlpC-CFP (CDC021) assembled into numerous long and curved filaments, often extending from one pole to the other (Figure 3B). However, some cells contained either a combination of filaments and foci or only foci. To gain more insight into the distribution of the AlpC-CFP filaments within the cell, Z-stacks were acquired. As shown in Figure 3C (and Movie S1), a number of long, curved filaments extending the length of the cell were observed, some filaments appearing to elongate close to the cell membrane. Thus, akin to actin and related cytoskeletal proteins AlpC can readily polymerizes forming filamentous structures *in vivo*. The catalytically inactive mutant AlpC^D301A^ also assembled into filaments *in vivo* when overproduced (CDC022, Supplementary Table S1) (Figure 3B). However, the frequency of filament formation was drastically reduced (1.6%, *n* = 750) and the morphology of the AlpC^D301A^ filaments was different from the wild type protein.

### AlpC filaments are dynamic

**Figure 4.**
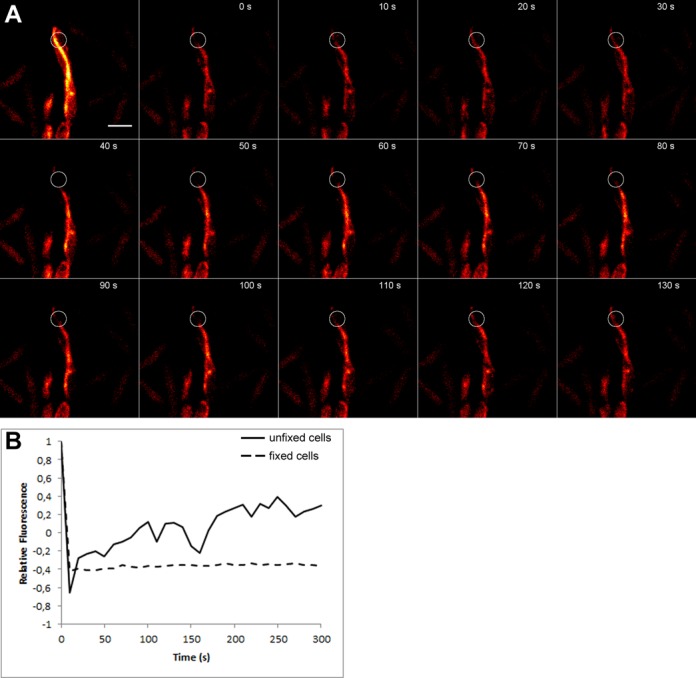
FRAP analysis of AlpC-mCherry. Cells encoding AlpC-mCherry were induced using 5 μM mitomycin C and imaged after 60 min of induction. Cells were grown on a BHI agar pad at 30°C. Part of the AlpC-mCherry filament was bleached using a 561 nm laser as described in Material and Methods section. Images were taken every 10 s. An assembly of still images acquired for 120 s is shown in (**A**). The full movie can be seen in the supplemental material (Movie S4). In a control experiment cells were fixed prior to bleaching and no recovery was observed (Supplementary Figure S2 and Movie S5). (**B**) FRAP curves for the AlpC-mCherry strain without fixing and with fixing prior to bleaching (fixed) are shown. Values are normalized for background fluorescence and intracellular mCherry fluorescence. The curve indicates recovery that is due to incorporation of unbleached AlpC-mCherry.

*In vivo*, filaments of the actin-like protein ParM involved in plasmid partition were shown to be extremely dynamic, undergoing bursts of rapid growth and catastrophic decay (59). Although such dynamic instability is not a distinguishing feature of actin and actin-like proteins, filament dynamics is essential for protein function. To ascertain if *C. glutamicum* AlpC is also dynamic, time-lapse analysis was carried out. For this purpose a strain harboring a plasmid-encoded AlpC-CFP translational fusion was used (CDC021, Supplementary Table S1). Cells were grown in BHI medium, induced with 0.5 mM IPTG for 1 h prior to microscopic analysis. Cells were placed on a microscopic slide coated with 1% agarose in BHI. Images were acquired at 5 s intervals for a total of 60 s. The time lapse analysis revealed that AlpC-CFP (CDC021) filaments are indeed dynamic (Figure 3D and Movie S2). While some filaments appear to move along the membrane, other filaments curl into the cytoplasm. *In vivo*, filament assembly of the catalytically inactive mutant (AlpC^D301A^-CFP, CDC022) is not completely abolished, however the frequency of filament formation is greatly reduced (Figure 3B). The AlpC^D301A^-CFP filaments are more stable and, hence less dynamic than wild-type AlpC filaments (Figure 3E, Movie S3). Filament dynamics were further studied using fluorescence recovery after photobleaching (FRAP) experiments. For this purpose, we constructed a strain harboring a plasmid-encoded AlpC-mCherry under control of the native promoter. This system allows for moderate AlpC-mCherry overproduction. The AlpC-mCherry strain was necessary for FRAP experiments because the natural level of AlpC leads to shorter filaments which will be bleached almost entirely and hence do not allow for FRAP analysis. AlpC-mCherry induction was achieved by addition of 5 μM mitomycin C. Readily, cells produced long, curved AlpC-mCherry filaments (Supplementary Figure S1). Parts of these filaments were bleached using the 561 nm laser and a subsequent time lapse series with image acquisition at 10 s intervals revealed rapid recovery of the AlpC-mCherry (Figure 4A and B, Movie S4). We controlled for dark-state-reversal of the mCherry fluorophore by fixing the cells with 1% formaldehyde. Subsequent FRAP experiments did not reveal any recovery of the mCherry fluorophore (Supplementary Figure S2, Movie S5), suggesting that the fast initial recovery is not a result of photoswitching, but reflects the true dynamics of the AlpC protein.

### Identification of an AlpC adaptor protein

**Figure 5.**
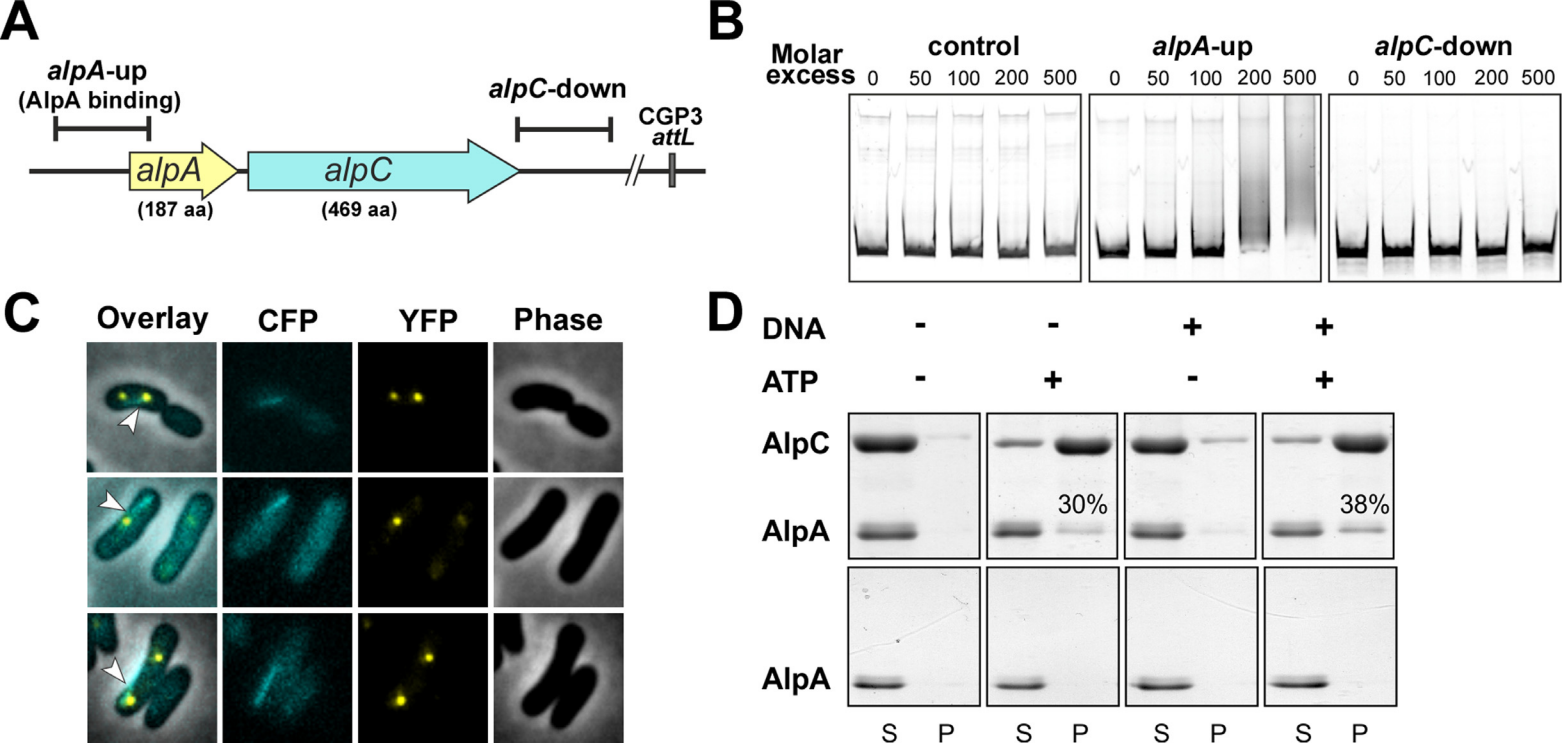
Characterization of the putative adaptor protein AlpA (cg1891) (**A**) Genomic localization of the AlpA binding region. The DNA fragment tested for AlpA-binding is located upstream of *alpA;* 110 bp downstream of the transcriptional start site of *alpA*. (**B**) EMSA studies of AlpA with potential DNA target regions. For DNA–protein interaction studies, DNA fragments (500 bp) of the up- and downstream region of the putative operon *alpA–alpC* were incubated with different molar ratios of AlpA (0-, 200- and 500-fold molar excess of AlpA). The promoter region of cg2036 served as control. (**C**) Co-visualization studies of eCFP-AlpC and AlpA-eYFP. The expression of both proteins was induced with mitomycin C for 2 h prior to localization analysis (**D**) AlpA interacts with AlpC *in vitro*. Purified AlpA and AlpC were incubated with ATP, DNA or both ATP and DNA. The supernatant and pellet fractions were separated by centrifugation. Both fractions were analyzed by SDS-PAGE and visualized by Coomassie staining. In the absence of AlpC, AlpA does not sediment, irrespective of the presence of ATP and/or DNA (lower panel). AlpA co-sedimentes with AlpC in the presence of ATP (30%) or ATP and DNA (38%). S, supernatant; P, pellet. The amount of sedimented AlpA protein was quantified by using ImageQuant TL software (GE Healthcare).

Actin-like proteins encoded on plasmids are often co-transcribed with an adaptor protein, which connects the actin filament with the plasmid DNA. In many cases, the adaptor protein stimulates or stabilizes the actin filaments (20,24,60,61). AlpC is encoded in a putative operon together with Cg1891, a protein of unknown function (Figure 1A). We postulated that Cg1891 could be an adaptor that couples phage DNA to AlpC filaments. Hence, we renamed cg1891 to *alpA* (A for adaptor). To test whether AlpA binds to specific DNA regions, purified His_10_-AlpA protein was analyzed by electrophoretic mobility shift assays (EMSA). We tested DNA fragments covering the up- and downstream region of the putative *alpA*–*alpC* operon (Figure 5A). The upstream region was specifically bound by AlpA, *in vitro* (Figure 5B). A smear of shifted DNA rather than a clear band was observed, indicating an oligomerization of the protein along the DNA, presumably at more than one binding site (62). The downstream region and the control DNA fragment (upstream of the CGP3 gene cg2040) showed no binding of AlpA.

**Figure 6.**
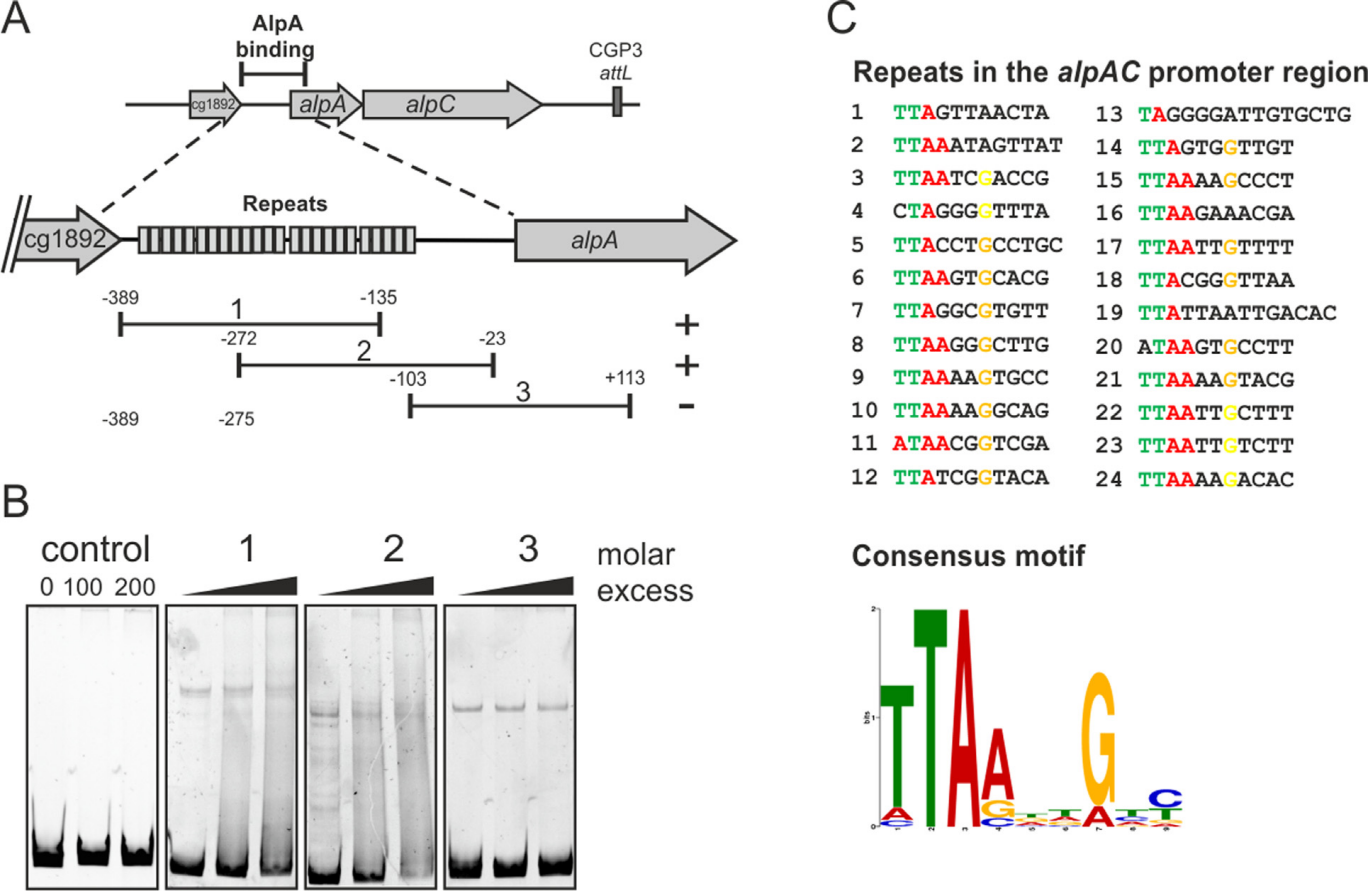
AlpA binds to conserved repeats in the *alpAC* promoter region. (**A**) Overview of the tested sub-fragments covering the upstream promoter region of *alpAC*. The conserved repeats are indicated as boxes. (**B**) Corresponding EMSA study to (A). To test for AlpA–DNA interaction, the DNA fragments were incubated with varying molar excess of AlpA (0-, 200- and 500-fold molar excess of AlpA; see supplemental material). (**C**) Conserved repeats in the *alpAC* promoter region. A consensus motif was derived using the MEME suite software (http://meme.nbcr.net/meme/).

Analysis of the *alpAC* promoter region revealed the presence of 24 conserved DNA repeats (designated as *alpS*) (Figure 6A). EMSA studies with purified AlpA were carried out to determine if AlpA binds to DNA fragments covering these repeats (Figure 6B). As outlined in Figure 6A, DNA fragments covering various portions of the *alpAC* promoter region were used for EMSA analysis. Both sub-fragments 1 and 2, which covered the majority of the conserved DNA repeats of the promoter region, displayed AlpA binding. The third sub-fragment and the control DNA, both of which lack DNA repeats, do not appear to be bound by AlpA. Using the MEME suite software a consensus motif was derived (TTAAnnG), which revealed five highly conserved bases within the *alpS* motif (Figure 6C, and Supplementary Figure S3). However, this DNA motif is not unique to the *alpAC* operon. In comparison to the whole genome, the *alpS* motif is more concentrated at the *alpAC* operon. These results suggest that AlpA binds to conserved DNA repeats, *alpS*, of the promoter region, however analysis of the exact mechanism will require further studies.

### Co-localization of AlpA and AlpC *in vivo*

**Figure 7.**
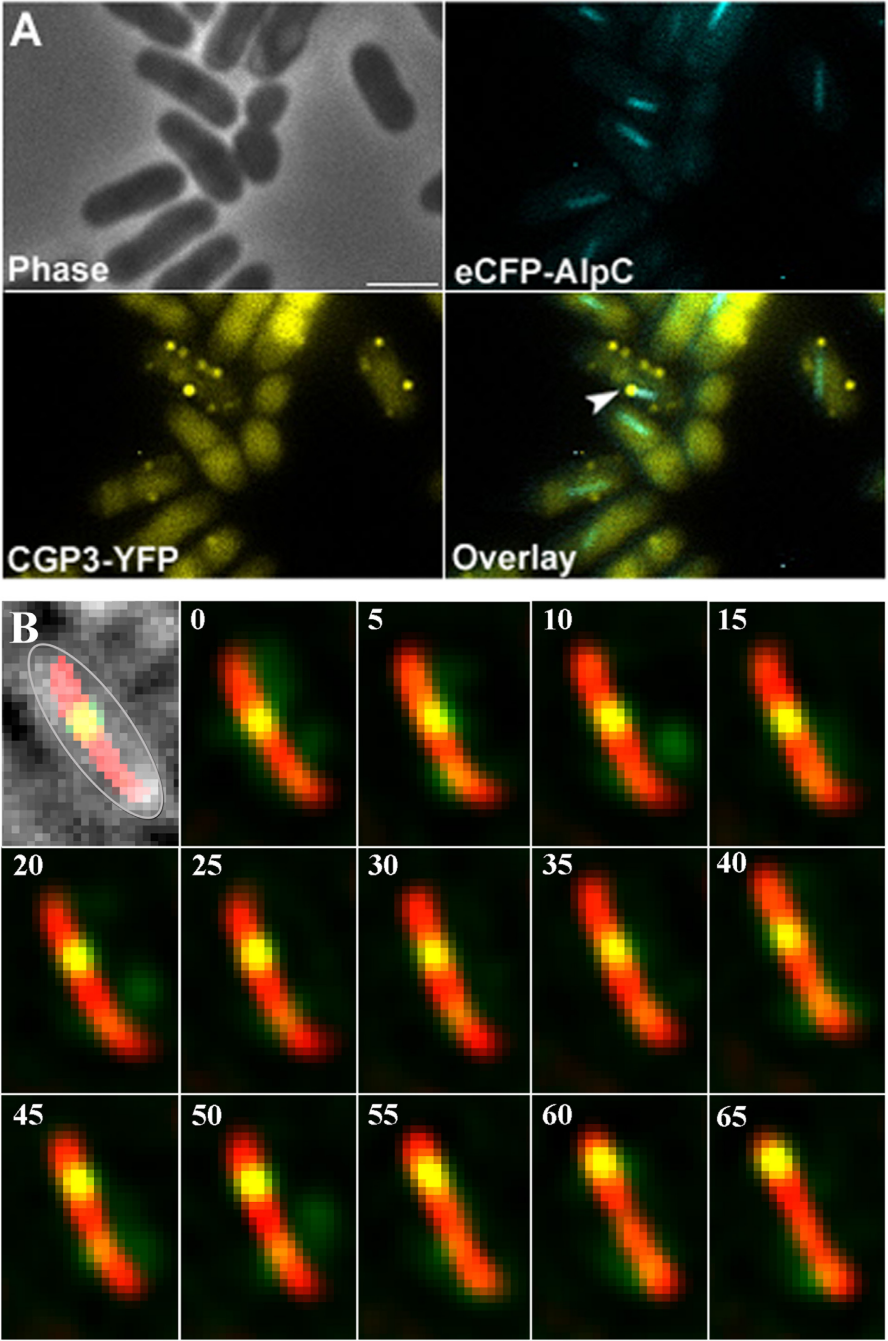
Co-visualization of eCFP-AlpC and induced CGP3 prophage (CGP3- YFP). (**A**). Cells expressing eCFP-AlpC as a single copy from the native promoter and a YFP labeled CGP3 DNA were treated with mitomycin C for 3 h to induce prophage excision. In the examples shown, the tip of an AlpC filament is connected to a CGP3 phage DNA molecule (arrowheads). (**B**) Time lapse analysis of AlpA-eYFP and AlpC-mCherry. Cells were treated with mitomycin C prior to microscope analysis. Images were acquired every 5 s for a total of 65 s (as indicated on the upper left corners). The first inset shows an overlay of the phase contrast, yellow and red channels, and the cell is outlined in gray. Within the time frame of the time lapse analysis AlpA-eYFP moves from the midcell region along the AlpC-mCherry filament to the cell pole (see also Movie S6). Scale bar, 2 μm.

To support our hypothesis that AlpA is an adaptor protein linking CGP3 DNA to the AlpC filament, localization of both proteins was studied *in vivo*. For this purpose, an in-frame deletion of *alpA* was constructed in the background of strain CDC020, in which *alpC* was replaced by allelic exchange with e*cfp-alpC*. Deletion of *alpA* had no influence on the formation of eCFP-AlpC filaments. AlpC also assembles into filaments when expressed in *E*. *coli* in the absence of AlpA (data not shown). To generate a strain that allows co-visualization of both AlpA and AlpC the *alpA* gene was fused to *eyfp* and cloned under control of the native promoter in the Δ*alpA alpC*::*ecfp*-*alpC* background (ATCC 13032 *ΔalpA alpC::ecfp-alpC* P*_alpA_-alpA-eyfp*, Supplementary Table S1). Fluorescence microscopy revealed foci of AlpA-eYFP, which formed upon induction of CGP3 by addition of mitomycin C. Frequently, one to two foci of AlpA-eYFP per cell were observed at various positions; in a few cases up to four foci were detected in single cells. Remarkably, AlpA-eYFP foci were observed at the tips or aligned with AlpC filaments (Figure 5C). In 78% (*n* = 37) of cells at least one AlpA focus was found to be associated with an AlpC filament. Co-localization was further confirmed with plasmid-encoded variants of AlpA-eYFP and AlpC-mCherry. In agreement with the genomic replacement, co-localization of AlpA-eYFP foci and AlpC-mCherry filaments was observed in 91% of the cells (*n* = 116, Supplementary Figure S1). In both experiments, the angle of the filaments and the position of the putative adaptor protein foci were, however, variable. The co-localization of AlpA and AlpC suggested that AlpA might move along the preformed AlpC filament track. To further reinforce this idea and that AlpA is AlpC-CGP3 adaptor protein, live cell time lapse co-visualization of AlpA-eYFP and AlpC-mCherry was carried out. Again, prior to microscopic analysis, cells were treated with mitomycin C to induce the CGP3 prophage. Time lapse analysis revealed that the AlpA-eYFP foci move along the AlpC-mCherry filament (Figure 7B and MovieS6).

### *In vivo* co-visualization of the CGP3 viral DNA and AlpC filaments

The CGP3 prophage and eCFP-AlpC (CDC020), where AlpC is expressed at the physiological level, were co-visualized *in vivo* by fluorescence microscopy. Visualization of CGP3 DNA was carried out as described previously (44). Basically, an array of *tetO* operator regions of transposon Tn10 was integrated into an intergenic region within the CGP3 prophage region. Co-expression of plasmid-encoded *yfp-tetR* allowed direct visualization of the prophage region. Prophage induction was triggered by mitomycin C addition. In many cells an increased number of foci corresponding to CGP3 DNA were visible (44). In these cells, eCFP-AlpC filaments or foci were readily observed. The eCFP-AlpC foci often co-localized with the CGP3 prophage foci (33%, *n* = 56) (Figure 7A). In many cases cells containing an AlpC filament had CGP3 DNA at the filament end. We noticed that most AlpC filaments point in an angle to the membrane and seem to push the phage DNA towards the cell membrane. Indeed, phage DNA is mostly found at the cell membrane (Figure 7A).

### AlpA interacts with AlpC *in vitro*

The binding of AlpA to phage DNA as well as the co-localization of AlpA foci with AlpC filaments, suggest a direct interaction of AlpAC, coupling the CGP3 DNA with the AlpC filament. To test this possibility further, we carried out co-sedimentation assays with AlpA and AlpC. While AlpC exhibits a nucleotide dependent sedimentation behavior, AlpA did not sediment after incubation with ATP, DNA or a combination of ATP and DNA (Supplementary Figure S4). When AlpA and AlpC were incubated in the presence of ATP or ATP and DNA, a significant fraction (30% and 38%, respectively) of AlpA protein co-sedimented with AlpC (Figure 5D). BSA served as a control protein, which did not co-sediment with AlpC (Supplementary Figure S4). Further support for a direct interaction between AlpA and AlpC filaments is provided by a super shift experiment where oligomerization of AlpC in the presence of ATP led to a super shift of AlpA-bound *alpS* DNA (Supplementary Figure S5).

### AlpA and AlpC are required for efficient CGP3 replication

**Figure 8.**
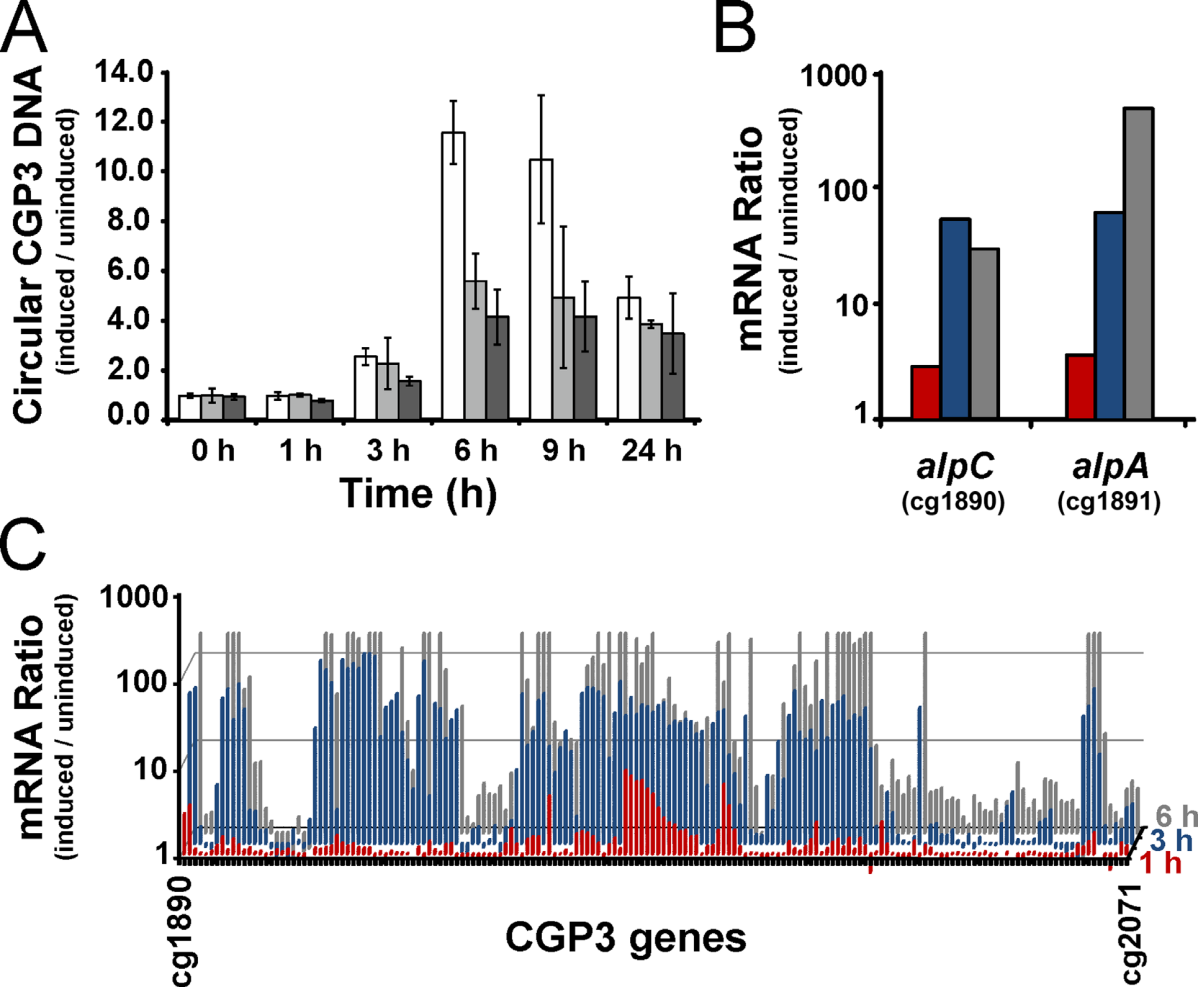
Impact of AlpC on CGP3 replication. (**A**) The relative amount of circular phage CGP3 DNA was quantified by qPCR in *C. glutamicum* wild type (white) and Δ*alpC* (light gray), and Δ*alpA (dark gray)* upon treatment with 0.6 μM mitomycin C. Shown are average values with standard deviation of three independent biological replicates. (**B**) Time course of *alpC* and *alpA* expression upon prophage induction triggered by the addition of 0.6 μM mitomycin C. Shown is the mRNA ratio of cells treated with mitomycin C *versus* untreated cells 1 (red), 3 (blue) and 6 h (gray) after mitomycin C addition analyzed by DNA microarrays. (**C**) Time course of the mRNA ratio of the whole CGP3 gene region after addition of mitomycin C (as described in B).

To investigate the physiological role of the actin-like protein AlpC and the adaptor protein AlpA, and respective roles in the replication and/or segregation of the prophage CGP3, in-frame deletion mutants lacking the *alpC* and *alpA* gene were constructed. Deletion of either *alpC* or *alpA* has no recognizable growth phenotype. In order to analyze the impact of AlpC and AlpC on CGP3 replication, *C. glutamicum* was treated with mitomycin C to induce prophage excision and replication. The intracellular amount of circular phage DNA significantly increased and reached ∼10-fold induction in mitomycin C treated cells after 6 h. Quantification of the intracellular amount of circular CGP3 DNA by qPCR in wild type and Δ*alpC* strains revealed a similar progression (Figure 8A). However, the maximal amount of circular CGP3 DNA was ∼2-fold reduced in Δ*alpC* cells compared to wild type. As observed for Δ*alpC*, the maximal amount of phage DNA was ∼2-fold reduced in Δ*alpA*. Deletion of the CGP3 encoded cg2040, a Cro/CI type regulator, did not alter the amount of circular phage DNA (data not shown). A complementation strain encoding AlpC-CFP had no significant difference to wild type, indicating that the fusion construct is functional (data not shown). The number of induced CGP3 phages was also quantified by means of fluorescent micrsocopy, *in vivo*. Thereby, the *alpC* gene was deleted in the ATCC 13032::pLAU44-CGP3-Spec strain background. In the absence of *alpC* (strain CDC024), the average number of CGP3 per cell was reduced compared to WT cells (1.9% and 2.9%, respectively (*n* ≥ 210)) (Supplementary Figure S6). These results indicate that AlpC is directly influencing CGP3 replication *in vivo*.

Transcriptome analysis revealed that both genes, *alpC* and *alpA*, are among the early genes of CGP3 induced upon treatment with mitomycin C (Figure 8B). The expression of *alpC* and *alpA* was 3–4-fold up-regulated 1 h after induction, whereas the majority of CGP3 genes showed an increased mRNA level after 3–6 h (Figure 8C). Further genes showing a >4-fold upregulation after 1 h include cg1962 and cg1977, encoding hypothetical proteins, as well as cg1996–1998 encoding a restriction modification system (*cglIMR*). Remarkably, cg1977 and the operon cg1996–1998 are both under direct control of LexA itself, explaining their high induction upon addition of mitomycin C. Altogether, early expression of *alpC* and *alpA* in the course of CGP3 induction and the reduced level of circular CGP3 DNA in the two deletion mutants emphasizes the participation of AlpC and AlpA in the early stages of phage induction and/or synthesis, such as replication of phage DNA.

## DISCUSSION

Virus particles can hijack the host cell cytoskeleton for active movement within the infected cells. Crucial for this is a dynamic assembly of a scaffold that can either serve as a molecular track or generate an intrinsic movement. Examples of such cytomotive filaments are prokaryotic actin-like proteins. Actin and actin-related proteins share limited sequence and structural similarity (33,34,63,64). The formation of dynamic filamentous structures *in vivo* is common to both eukaryotic and prokaryotic actin homologs. In *C. glutamicum*, AlpC readily assembles into long curved filaments when overproduced (Figure 3B and C). At physiological concentration, AlpC assembled into short straight filaments, in addition to formation of compact foci (Figures 5C and 7A). The dynamics of actin-like filaments varies and is often linked to the mode of subunit assembly, stability and function of the protein in question (65). ParM, for example, exhibits extreme dynamic instability, displaying bursts of growth followed by rapid decay (24,59,66). Stabilization of the ParM filament, which comprises of two antiparallel filaments, requires that one end of each ParM filament, is capped with a ParR bound plasmid (60,67). Thus, the dynamic instability of the ParM filament is intrinsic to the mode of action in plasmid segregation. Similarly, the mode of action of Alp7A filaments in plasmid segregation requires that both ends of the filament are capped with a plasmid (56). The actin-like protein AlfA assembles into stable long-lived filaments by addition of subunits to one end of the filament only (61). Although the mechanism by which Alp7A segregates plasmids is not well understood, it differs from ParM mediated segregation (20,61). AlpC filaments exhibited a dynamic behavior (Figure 4A and Movie S4); however, unlike ParM and Alp7A dynamic instability was not observed (24,56,59,66).

Initially, we speculated that AlpC might function to actively segregate excised prophage DNA, akin to plasmid segregation systems. However, the genetic organization of the *alpAC* operon differs (Figure 1A) from known chromosome/plasmid segregation loci (ParM, ParR), which encode a motor protein upstream of the DNA-binding adaptor protein. An example of a similar genetic organization is found in Gordonia sp. KTR8, where two gene homologs to *alpA* and *alpC* are found on the plasmid pGKT2 (Figure 1A). In this case, the two proteins might play a role in plasmid segregation. In cells that contained multiple CGP3 foci and AlpC filaments, interaction between AlpC filaments and at least one phage DNA molecule was observed (Figure 7A and B), arguing against a role in segregation of two phage genomes into daughter cells. In the light of recent studies, which indicate that induction of the cryptic prophage CGP3 leads to cell death (Pfeifer & Frunzke, submitted for publication) a function of AlpAC in the segregation of CGP3 DNA into dividing daughter cells appears unlikely. Although CGP3 represents a cryptic, degenerated prophage element several functions, including the excision from the host genome, the SOS-dependent control of the lysogenic switch and the killing of the host cell, represent conserved processes of a lytic cycle (68).

The DNA of the induced prophage often accumulated at the cell membrane (Figure 7A and B). There is an increasing amount of evidence suggesting that plasmid and phage replication occurs at a specific subcellular localization, e.g. at the membrane (5,39,41,69). The *B. subtilis* phage φ29 serves as a model for the study of phage replication. During the early stages of infection phage replication takes place at the bacterial nucleoid and later in the infection cycle replication is redirected to the bacterial membrane (5,39,41,69). Here, we speculate that excised CGP3 phage DNA is directed to the membrane where additional replication occurs. Based on our data, we propose that circular CGP3 DNA is connected to AlpC filaments *via* the adaptor protein AlpA, which interacts with *alpS* repeats upstream of the *alpAC* promoter (Figures 5 and 6). Consistent with this idea, cells lacking AlpC or AlpA have a 2-fold decrease in phage copy number (Figure 8), which phenocopies the impact of a *phuZ* deletion on the replication of phage 201φ2-1 DNA (43) and, thus, clearly suggests a concerted role of AlpAC in CGP3 phage replication.

Recently, reports on phage-encoded tubulin homologs have been published (43,70). Both reports describe that phage-encoded PhuZ (43) and TubZ (70) are required for correct phage placement in the infected host. Interestingly, the two reports arrive at different conclusions. PhuZ is encoded in the *Pseudomonas* phage 201φ2-1 and forms GTP hydrolysis dependent dynamic filaments *in vivo* and *in vitro* (43). These filaments traverse the entire cell and are thought to assemble phage particles in the center of the cell. However, PhuZ was expressed from an inducible promoter. Here, we have observed a similar polymerization behavior of AlpC when it was overproduced in *C. glutamicum*, but got a different picture when *alpC* was expressed from the native promoter. Phage replication was also impaired in a PhuZ mutant background, suggesting that proper segregation is important for phage replication. The authors arrive at the conclusion that PhuZ forms a spindle-like apparatus that positions the virus particles in the cell center (43). Simultaneously, a TubZ homolog in the *Clostridum botulinum* phage c-st was shown to behave like a classical type II segregation system. TubZ binds to the centromeric region of the phage DNA *via* the adaptor protein TubR (70). Superficially, the c-st TubZ and AlpC share a similar mechanism, in particular since both are encoded by prophages that replicate as plasmid-like entities. However, close inspection of the results that we describe here, make it unlikely that the short AlpC filaments observed after induction of phage replication would be suited to segregate plasmid DNA into separating daughter cells. Rather, our data are consistent with AlpC guiding CGP3 DNA to the cell membrane, where replication likely occurs. Fluorescent co-localization of AlpC filaments and viral DNA show that phage DNA foci are often found in numerous copies at the membrane, while AlpC filaments only attach to single CGP3 molecules. In this aspect AlpC might resemble the role of eukaryotic F-actin in the anterograde transport of herpes-like viruses (46,47,71). The transport brings virus particles to the cell membrane. Actin plays, in fact, a prominent role as a host factor for viral replication in eukaryotes. Actin is not only required for anterograde transport, but also involved in uptake, retrograde transport to the nucleus, replication and long-range spread (47). This is in line with observations that link the *B. subtilis* actin homolog MreB with replication of phage φ29 (41). In the absence of MreB, phage polymerase and the putative membrane anchor p16.7 are delocalized. Consequently, phage replication was reduced. It may, therefore, be advantageous for phages replicating in bacteria lacking an endogenous actin cytoskeleton to encode this cytoskeletal element within their own genome. A unifying theme is that phages with large genomes seem to rely on cytoskeletal filaments for intracellular movement. Thus, it is plausible that the connection of actin and viral replication is ancient (72) and that the general principle is well conserved even between bacteria and phages.

## SUPPLEMENTARY DATA

Supplementary Data are available at NAR Online.

SUPPLEMENTARY DATA
