# Supplementary material for: A prophage-encoded actin-like protein required for efficient viral DNA replication in bacteria
Source: Nucleic Acids Res. 2015 Apr 27;43(10):5002–16. doi: 10.1093/nar/gkv374 (PMC4446434; doi:10.1093/nar/gkv374)
Supplement: SUPPLEMENTARY DATA [file supp_43_10_5002__index.html]

A prophage-encoded actin-like protein required for efficient viral DNA replication in bacteria — A prophage-encoded actin-like protein required for efficient viral DNA replication in bacteria — SUPPLEMENTARY DATA 

# A prophage-encoded actin-like protein required for efficient viral DNA replication in bacteria

## SUPPLEMENTARY DATA

**Files in this Data Supplement:**

- SUPPLEMENTARY DATA
- SUPPLEMENTARY DATA
- SUPPLEMENTARY DATA
- SUPPLEMENTARY DATA
- SUPPLEMENTARY DATA
- SUPPLEMENTARY DATA
- SUPPLEMENTARY DATA
