# Supplementary material for: A prophage-encoded actin-like protein required for efficient viral DNA replication in bacteria
Source: Nucleic Acids Res. 2015 Apr 27;43(10):5002–16. doi: 10.1093/nar/gkv374 (PMC4446434; doi:10.1093/nar/gkv374)
Supplement: SUPPLEMENTARY DATA [file supp_gkv374_nar-00271-v-2015-File010.docx]

# Supplemental Tables, Figures and Movies

A prophage encoded actin-like protein required for efficient viral DNA replication in bacteria

Catriona Donovan^1,3,*^, Antonia Heyer^2,*^, Eugen Pfeifer, Tino Polen^2^, Anja Wittmann^1^, Reinhard Krämer^1^, Julia Frunzke^2,‡^, & Marc Bramkamp^1,3‡^

^1^ Department of Biology I, Ludwig-Maximilians-University Munich, Großhaderner Str. 2-4, 82152 Planegg-Martinsried, Germany.

^2^Institut für Bio- und Geowissenschaften, IBG-1: Biotechnologie, Forschungszentrum Jülich, D-52425 Jülich, Germany;

^3^ Institute for Biochemistry, University of Cologne, Zülpicherstr. 47, 50674 Cologne, Germany.

**Strain construction**

The in-frame deletion mutant of the *alpC* gene (cg1890) was constructed *via* the two-step homologous recombination procedure as described previously ([1](#_ENREF_1)). The *alpC* up- and downstream regions were amplified using the oligonucleotide pairs DalpC-1/DalpC-2 and DalpC-3/DalpC-4. The resulting PCR products were used as a template for an overlap extension PCR with the oligonucleotides DalpC-1/DalpC-4. The purified PCR product of approximately 1 kb was digested with EcoRI and BamHI and cloned into the pK19*mobsacB* vector*.* The resulting plasmid pK19*mobsacB*-Δ*alpC* was used for performing an allelic exchange by homologous recombination ([1](#_ENREF_1)) in the chromosome of *C. glutamicum* ATCC 13032 resulting in the mutant strain *C. glutamicum* Δ*alpC*.

The strain expressing *ecfp*-*alpC* (CDC020) at physiological concentration was generated by integrating *ecfp* upstream of and in-frame with *alpC*. As describe above, a two-step homologous recombination procedure was used ([1](#_ENREF_1)). The *ecfp* gene was PCR amplified using the primer pair eCFP-SalI-F/eCFP-XbaI-R. The resulting PCR product was restriction digested with SalI and XbaI and ligated into an identically digested pK19*mobsacB* vector, placing *ecfp* in the middle of the multiple cloning site. The region upstream of the *alpC* start codon and the first 0.5 kp of *alpC* were amplified using primer pairs Alp-up-Hind-F/Alp-up- Sal-R and Alp-D-Xba-F/Alp-D-Bam-R, respectively. The resulting PCR products were digested with HindIII/SalI and XbaI/BamHI, respectively and sequentially ligated into a pK19*mobsacB*-*ecpf* vector. This vector was used for allelic replacement at the *alpC* locus, integrating *ecfp* at the 5’ end of *alpC*. Chromosomal integration was confirmed by PCR.

To generate a strain overproducing *alpC*-*cfp* (CDC021), *cfp*, including the stop codon, was amplified using primer pair CFP-SacI-F/cfp EX2 Eco R ms. The resulting PCR product was restriction digested with SacI and EcoRI and ligated into an identically treated pEKEx2 vector. Subsequently, *alpC*, lacking the stop codon, was amplified using primer pair AlpC-SalI- F/ AlpC-BamHI-os-R, restriction digested with SalI and BamHI and ligated into the pEKEx2-*cfp* vector. The resulting vector (pCD129) was transferred into *C. glutamicum* resulting in extrachromosomal, IPTG-inducible expression of *alpC*-*cfp*.

To generate a strain expressing *alpC^D301A^*-*cfp* (CDC022), pCD129 was used as a template and subjected to site directed mutagenesis using primer pair 1890-D301A-F/1890-D301A-R, giving rise to vector pCD130. Plasmids were verified by sequencing. The resulting vector was transferred into *C.* *glutamicum*, giving rise to strain.

For plasmid-encoded expression of *alpC*-*mCherry*, the *alpC* orf was fused to the gene encoding the mCherry fluorescent protein and cloned into the vector pEC-XC99E under control of the native P*_alpAC_* promoter. The fragments were amplified using the primer pairs PalpA-F/PalpA-R, AlpC_F/AlpC_R and mCherry_F/mCherry_R and the resulting fragments were assembled using Gibson Assembly ([2](#_ENREF_2)).

For an in-frame deletion of *alpA* (cg1891) the plasmid pK19*mobsacB-ΔalpA* was constructed using the oligonucleotides DalpA-1/DalpC2 and DalpA-3/DalpA-4*.* For deletion of *alpA* in the strain CDC020 (ATCC 13032 Δ*alpA* *alpC*::*ecfp*-*alpC*), in which *alpC* is genomically replaced by *ecfp-alpC*, the flanking downstream region of *alpA* was amplified with the oligonucleotides DalpA-3 and DalpA-cfp-alpC-4. As a template DNA for this PCR served genomic DNA of strain CDC020. In an overlap extension PCR, the PCR products were combined by amplification with the oligonucleotides DalpA-1 and DalpA-4 or DalpA-cfp-alpA-4, respectively. After restriction with EcoRI and BamHI and ligation with pK19*mobsacB,* the plasmids pK19*mobsacB-ΔalpA* and pK19*mobsacB*-Δ*alpA*-*cfp-alp* were obtained. The construction of an in-frame deletion mutant of *alpA* and the deletion of *alpA* in the strain CDC020 were perform as described for Δ*alpC* resulting in the mutant strains Δ*alpA* and *ΔalpA alpC::ecfp-alpC.*

To localize AlpA in single cells, an AlpA-eYFP protein fusion was used. For this purpose, the natural promoter P*alpA* and *alpA*, excluding the stop codon, were amplified by PCR with the oligonucleotides pairs PalpA-BamHI-fw and alpA-link-rv. To amplify *eyfp* the oligonucleotides eyfp-link-fw and eyfp-SalI-rv were used, thereby adding the sequence 5’- GGCGCTGCTGGC-3’ in front of *eyfp* as a linker sequence. Both PCR products were combined in an overlap-extension PCR with the oligonucleotides PalpA-BamHI-fw and eyfp- SalI-rv and digested with BamHI and SalI. Subsequently, the product was ligated into pJC1, resulting in the plasmid pJC1-*PalpA-alpA*-*eyfp*. For co-localization studies the strain ATCC 13032 Δ*alpA* *alpC*::e*cfp-alpC* was transformed with the plasmid resulting in strain ATCC 13032 *ΔalpA alpC::ecfp-alpC* P*_alpA_-alpA-eyfp*.

For co-visualization of eCFP-AlpC filaments and CGP3 prophages (CDC023), strain CDC020 was used as the background strain and the CGP3 prophage was tagged as described previously ([3](#_ENREF_3)).

For co-visualization of AlpC-mCherry and AlpA-eYFP, wild type *C. glutamicum* was co-transformed with plasmids pJC1-*PalpA-alpA*-*eyfp* and pEXC-XC99E-P*alpC*-*alpC*-*mCherry* resulting in strainATCC 13032 *alpC*-*mCHerry* *alpA*-*eyfp*.

For heterologous overexpression of His_10_-AlpC the *alpC* gene (cg1890) was PCR amplified using primers AlpC-et-F and AlpC-et-R, restriction digested with XhoI and BamHI and ligated into pET16b (Novagen). The resulting plasmid (pCD115) was transformed into BL21(DE3) pLysS for heterologous protein production. Site directed mutagenesis was employed to generate pCD116 (His_10_-AlpC^D301A^). For heterologous overexpression of AlpA the gene cg1891 (*alpA*) was amplified with the oligonucleotides alpA-fw and alpA-rv. The PCR product and the plasmid pET-TEV were digested with NdeI and EcoRI and ligated. Thereby, the sequence of a His_10_-tag was fused to the 5’- end of *alpA*, resulting in the plasmid pET-TEV-*alpA*. Subsequently, *E. coli* BL21(DE3) was transformed with the plasmid for heterologous protein production.

**Table S1:** Bacterial strains, plasmids and oligonucleotides.

| **Strains** | **Relevant characteristics** | | **Source or reference** | |
| --- | --- | --- | --- | --- |
| ***C. glutamicum* ATCC 13032** | Biotin-auxotrophic wild type | | ([4](#_ENREF_4)) | |
| **ATCC 13032 Δ*alpC*** | In-frame deletion of cg1890 (*alpC*) | | This study | |
| **CDC020** | ATCC 13032 with an in-frame allelic replacement of *alpC* to *ecfp-alpC* | | This study | |
| **CDC021** | ATCC 13032, IPTG-inducible extra-chromosomal copy of *alpC-cfp*, Kan^r^ | | This study | |
| **CDC022** | ATCC 13032, IPTG-inducible extra-chromosomal copy of *alpC^D301A^-cfp*, Kan^r^ | | This study | |
| **ATCC 13032::pLAU44-CGP3-Spec** | ATCC 13032 derivative containing plasmid pLAU44-CGP3-Spec integrated into the cg1905-cg1906 intergenic region | | ([3](#_ENREF_3)) | |
| **CDC023** | ATCC 13032 derivative containing plasmid pLAU44-CGP3-Spec integrated into the cg1905-cg1906 intergenic region in the strain CDC020 | | This study | |
| **CDC024** | ATCC 13032 derivative containing plasmid pLAU44-CGP3-Spec integrated into the cg1905-cg1906 intergenic region in the strain ATCC 13032 Δ*alpC* | | This study | |
| **ATCC 13032 Δ*alpA*** | ATCC 13032 with in-frame deletion of cg1891 (*alpA)* | | This study | |
| **ATCC 13032 *ΔalpA alpC::ecfp-alpC*** | ATCC 13032 with in-frame deletion of cg1891 (*alpA*) in the strain CDC020 with an allelic replacement of *alpC* to *ecfp-alpC* | | This study | |
| **ATCC 13032 *ΔalpA alpC::ecfp-alpC* P*_alpA_-alpA-eyfp*** | ATCC 13032 with in-frame deletion of *alpA* (cg1891) in the strain CDC020 with an allelic replacement of *alpC* to *ecfp*-*alpC* and plasmid-bound expression of *alpA*-*eyfp* (pJC1-P*_alpA_-alpA-eyfp*) | | This study | |
| **ATCC 13032 *alpC*-*mCherry*** | ATCC 13032 derivative containing plasmid pEXC-XC99E-P*alpC*-*alpC*-*mCherry* Cm^r^ | | This study | |
| **ATCC 13032 *alpC*-*mCherry alpA-eYFP*** | ATCC 13032 derivative containing plasmid pEXC-XC99E-P*alpC*-*alpC*-*mCherry* Cm^r^ and plasmid-bound expression of *alpA*-*eyfp* (pJC1-P*_alpA_-alpA-eyfp*) Kan^r^ | | This study | |
| ***E. coli* DH5α** | *supE44* Δ*lacU169* (ф80*lacZ*DM15) *hsdR17* *recA1 endA1 gyrA96 thi-1 relA1* | | Invitrogen | |
| ***E. coli* BL21 (DE3) pLysS** | F^–^ *ompT hsdS*(r_B_^–^ m_B_^–^) *gal dcm* λ(DE3) pLysS (Cam^r^ ) (λ(DE3): *lac*I, *lac*UV5-T7 gene 1, *ind*1, *sam*7, *nin*5 ) | | Promega | |
| **Plasmids** | **Relevant characteristics** | | **Source or reference** | |
| **pK19*mobsacB*** | Kan^r^; vector for allelic exchange in *C. glutamicum*; (pK18 *oriV_E.c_*_._, *sacB*, *lacZ*α) | | ([5](#_ENREF_5)) | |
| **pK19*mobsacB-ΔalpA*** | Kan^r^; pK19*mobsacB* derivative containing a crossover PCR product covering the up- and downstream regions of *alpA* (cg1891) | | This study | |
| **pK19*mobsacB-ΔalpA-ecfp-alpC*** | Kan^r^; pK19*mobsacB* derivative containing a crossover PCR product covering the flanking regions of *alpA* (cg1891) in strain CDC020 (*alpC* replaced by *ecfp*-*alpC*) | | This study | |
| **pK19*mobsacB-*Δ*alpC*** | Kan^r^; pK19*mobsacB* derivative containing a crossover PCR product covering the up- and downstream regions of *alpC* (cg1890) | | This study | |
| **pCD127** | Integration vector*, ori pUC, Kan^r^, mob sacB ecfp-alpC* | | This study | |
| **pEKEx2** | Kan^r^; *C. glutamicum*/*E. coli* shuttle vector for regulated gene expression (P_tac_, *lacI*^q^, pBL1 *oriV*_Cg_, pUC18 *oriV*_Ec_) | | ([6](#_ENREF_6)) | |
| **pCD129** | Kan^r^, P*_tac_ lacI*^q^ pBL1 *oriV_C_*_._*_g_*. pUC18 *oriV_E_*_._*_c_*, AlpC^+^-CFP | | This study | |
| **pCD130** | Kan^r^, P*_tac_ lacI*^q^ pBL1 *oriV_C_*_._*_g_*. pUC18 *oriV_E_*_._*_c_*, AlpC^D301A+^-CFP | | This study | |
| **pEKEx2-*yfp-tetR*** | Kan^r^, pEKEx2 derivative containing *yfp-tetR* under control of the P*_tac_* promoter | | ([3](#_ENREF_3)) | |
| **pET16(b)** | *bla PT7lac-10his lacI* | | Novagen | |
| **pCD115** | *bla PT7lac-10his-alpC lacI* | | This study | |
| **pCD116** | *bla PT7lac-10his-alpC^D301A^ lacI* | | This study | |
| **pET-TEV** | Kan^r^; pET28b derivative for overexpression of genes in *E. coli* (pBR322 *oriV_E.c._ , PT7, lacI*) | | ([7](#_ENREF_7)) | |
| **pET-TEV-*alpA*** | Kan^r^, pET-TEV derivative to overproduce AlpA with an N-terminal His_10_ tag | | This study | |
| **pJC1** | Kan^r^, Amp^r^, *E. coli* – *C. glutamicum* shuttle vector | | ([8](#_ENREF_8)) | |
| **pJC1-P*_alpA_-alpA-eyfp*** | Kan^r^, pJC1 derivative containing *alpA-eyfp*, encoding an AlpA-eYFP protein fusion under the control of the native promoter P*_alpA_* | | This study | |
| **pEC-XC99E** | *cat_I_*, *lacl^q^*, P*_trc_*, *rrnB* (T1 and T2), *oriV_E.c_*, *per* and *repA* (pGA1*)_C.g._,* - *E. coli* – *C. glutamicum* shuttle and expression vector with chloramphenicol resistance | | [([9](#_ENREF_9))] | |
| **pEC-XC99E-P*_alpA_*-*alpC*-*mCherry*** | Cm^+^, pEC-XC99E containing *alpC-mcherry*, encoding an AlpC-mcherry protein fusion under the control of the native promoter P*_alpA_* (used restriction site: *Pst*I, *Nde*I) | | This study | |
| **Oligonucleotide** | **Sequence (5´→ 3´) and properties^a^** | | |  |
| DalpC-1 | ATATATGAATTCTTGTTTGTCGCTGAATACGGTG (EcoRI) | | |  |
| DalpC-2 | *CCCATCCACTAAACTTAAACA*CACATTCACAGCGCTGGTCATAATC | | |  |
| DalpC-3 | *TGTTTAAGTTTAGTGGATGGG*CGCTCGATTGCAGCGAAAGCACG | | |  |
| DalpC-4 | TATATAGGATCCAGCGCGCCAAAGAAAACACAGAG (BamHI) | | |  |
| DalpA-1 | TATATAGAATTCCCATTTTCGGGGTGATGGTTAC (EcoRI) | | |  |
| DalpA-2 | *CCCATCCACTAAACTTAAACA*CGTGTCCTGTTTTTGAGCCATGTG | | |  |
| DalpA-3 | *TGTTTAAGTTTAGTGGATGGG*GATGTCATGGGCCAAGCGTTCG | | |  |
| DalpA-4 | TATATAGGATCCTCGTGCAGACAAGCTGCGCGTGCC (BamHI) | | |  |
| DalpA-cfp-alpC-4 | TATATAGGATCCGTCCTCCTTGAAGTCGATGCC (BamHI) | | |  |
| alpA-NdeI-fw | CGCCATATGGCTCAAAAACAGGACACGAC (NdeI) | | |  |
| alpA-EcoRI-rv | CCGGAATTCCTAGCGACCGAACGCTTGG (EcoRI) | | |  |
| PalpA-BamHI-fw | CGCGGATCCGTCATGGTGGGGCTCCATTAG (BamHI) | | |  |
| alpA-link-rv | *GCCAGCAGCGCC*GCGACCGAACGCTTGGCC | | |  |
| eyfp-link-fw | *GGCGCTGCTGGC*ATGGTGAGCAAGGGCGAGG | | |  |
| eyfp-SalI-rv | CGCGTCGACTTATCTAGACTTGTACAGCTCGTC (SalI) | | |  |
| alpA-up-fw | TGTCATGGTGGGGCTCCATTA | | |  |
| alpA-up-rv | TGTGTTTCCAAAAACCGCTCAAC | | |  |
| alpC-down-fw | AAGGACTTATGATTGCGGCAC | | |  |
| alpC-down-rv | CTGGGGGCTAGAGCGCGCCAA | | |  |
| Cg2036-up-fw | CACTTCCAAGGAAGATACACGC | | |  |
| Cg2036-up-rv | ATGTTAAGAGCGTAACGGATATCA | | |  |
| Phage-LC-for | CCCACGTTCACCCCACAAACG | | |  |
| Phage-LC-rev | CTAAAATGAAGCCATCGCGACC | | |  |
| ddh-LC-for | ACGTGCTGTTCCTGTGCATGG | | |  |
| ddh-LC-rev | GCTCGGCTAAGACTGCCGCT | | |  |
| Alp-up-Hind-F | CAGAAGCTTTGTGGGTGAAGGTACT (HindIII) | | |  |
| Alp-up-Sal-R | CAGGTCGACCTTGCGTGCTTTCGC (SalI) | | |  |
| Alp-D-Xba-F | CAGTCTAGATAATTAATACCTAGTT (XbaI) | | |  |
| Alp-D-Bam-R | CATGGATCCCCACTCATTACCGCC (BamHI) | | |  |
| eCFP-SalI-F | CATGTCGACATGGTGAGCAAGGGC (SalI) | | |  |
| eCFP-XbaI-R | CATCTCGAGCTTGTACAGCTCGTC (XbaI) | | |  |
| AlpC-SalI-F | CAGGTCGACATGACCAGCGCTGTGAAT (SalI) | | |  |
| AlpC-BamHI-os-R | CAGGGATCCCTTGCGTGCTTTCGCTGC (BamHI) | | |  |
| CFP-SacI-F | CAGGAGCTCATGGTGAGCAAGGGCGAG (SacI) | | |  |
| cfp EX2 Eco R mS | GCGGAATTCTTACTTGTACAGCTCGTC (EcoRI) | | |  |
| 1890-D301A-F | CGCCACACAATCGGTGTG**GCC**GTGGGTGAAGGTACTG | | |  |
| 1890-D301A-R | CAGTACCTTCACCCAC**GGC**CACACCGATTGTGTGGCG | | |  |
| AlpC-et-F | CAGCTCGAGATGACCAGCGCTGTG (XhoI) | | |  |
| AlpC-et-R | CAGGGATCCTTACTTGCGTGCTTTCGC (BamHI) | | |  |
| PalpA-F | **GCGGTATTTCACACCGCATATG**GGTGATGGTTACAGCATCGC | | |  |
| PalpA-R | **ATATCTCCTTCTTAAAGTCTA**TACATGTGTCGTGTCCTGTTTTTG | | |  |
| AlpC-F | **TAGACTTTAAGAAGGAGATAT**ATGACCAGCGCTGTGAATGTG | | |  |
| AlpC-R | **TCCTCGCCCTTGCTCACCAT**CTTGCGTGCTTTCGCTGCAAT | | |  |
| mCherry-F | | ATGGTGAGCAAGGGCGAG | | |
| mCherry-R | | **AACAGCCAAGCTTGCATGCC**TTACTTGTACAGCTCGTCCATGC | | |

^a^ In some cases oligonucleotides were designed to introduce recognition sites for restriction endonucleases (recognition sites underlined, restriction endonucleases indicated in parentheses) or complementary 21mer sequences for generating overlap PCR products (italics). Amino acid mutations on mutagenesis primers are indicated in grey boldfacing. In bold, overlaps for Gibson assembly are indicated ([2](#_ENREF_2)).

**Supplementary Figures**

**
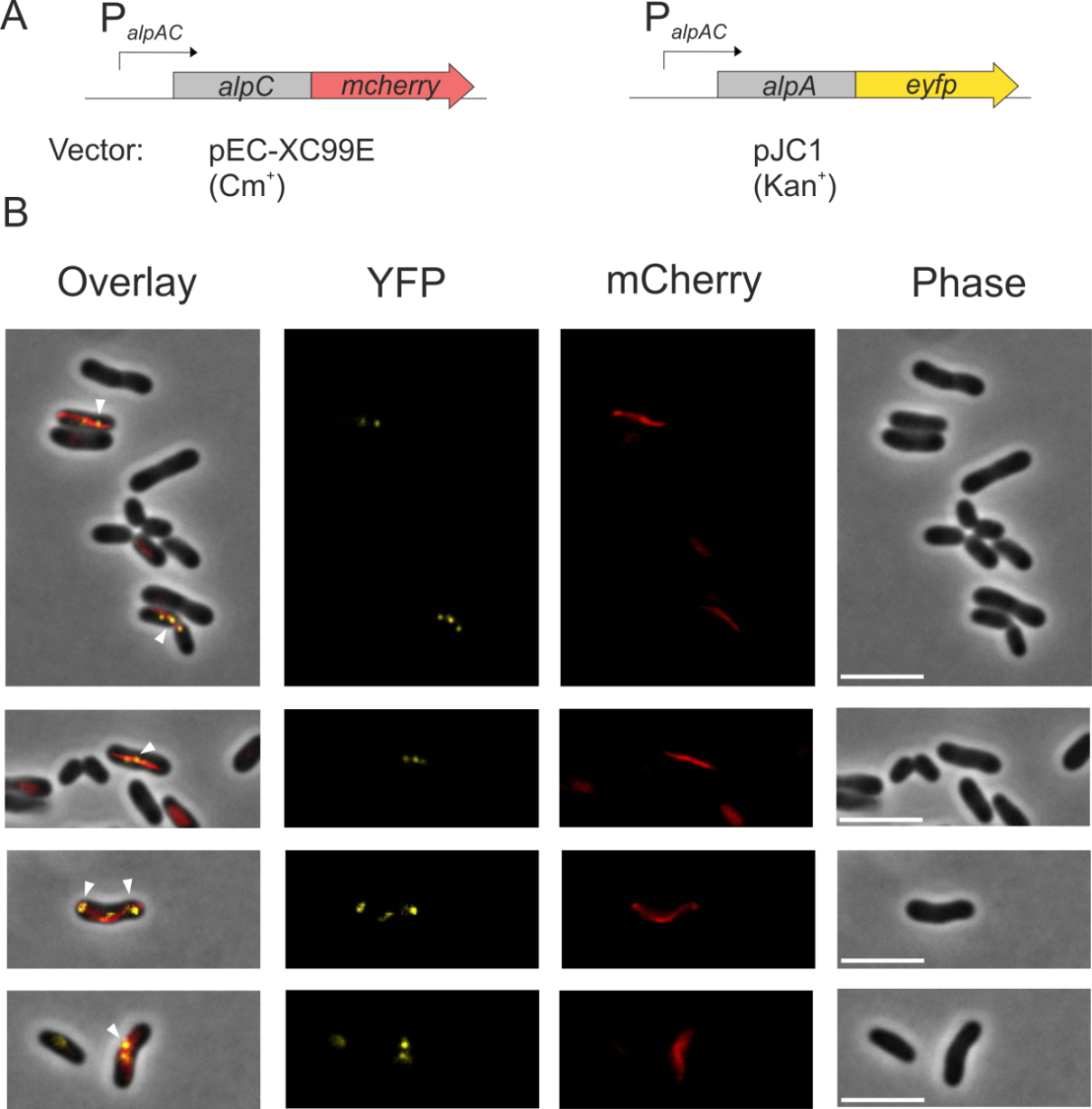
**

**Figure S1: Co-localization studies of AlpA and AlpC filaments after mitomycin C treatment.** **A:** Reporter constructs which were used to monitor localization of AlpA-eYFP and AlpC-mCherry in *C. glutamicum* ATCC 13032. B: Wild type cells of the strain ATCC 13032 containing both plasmids (pEC-XC99E-alpA-yfp and pJC1-alpC-mcherry) were grown in CGXII minimal media supplemented with 2% glucose, 10 µg/ml chloramphenicol and 25 µg/ml kanamycin to an OD_600_ of approximately three. Activation of the CGP3 prophage was induced by adding mitomycin C (0.6 µM). Three to five hours after induction pictures were taken by using fluorescence microscopy (length of the bar scale is 5 µm). Overall, co-localization of AlpA foci and AlpC filaments was observed in 91% of the cells (n=116).

**Figure S2: FRAP experiment to control for dark-state-reversal of mCherry fluorophore.** To control for dark-state-reversal of the mCherry fluorophore, cells expressing AlpC-mCherry were fixing with 1% formaldehyde for 30 minutes at room temperature prior to analysis. FRAP analysis was carried out and evaluated as described in materials and methods. After bleaching a region in the cell, no recovery of mCherry was detected.


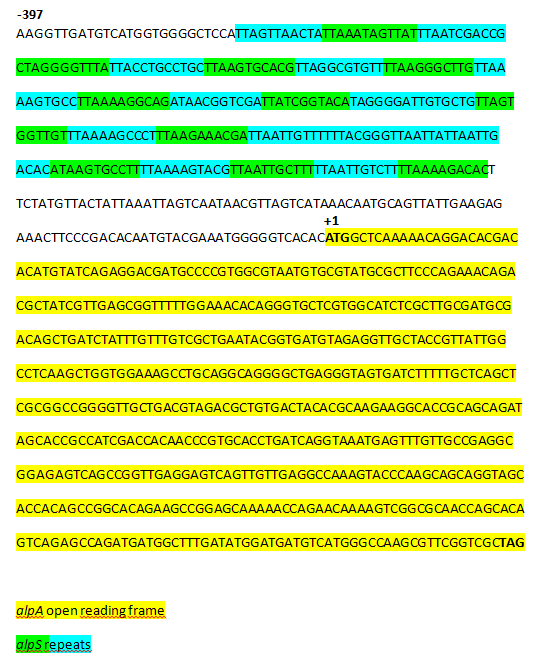


**Figure S3: Conserved *alpS* repeats in the *alpAC* promoter region.** Shown are 397 bases of the *alpAC* operon followed by the *alpA* orf (yellow). The *alpS* repeats, which were shown to be bound by AlpA *in vitro*, are highlighted in green and blue.

–––

**Figure S4: Co-sedimentation analysis of AlpA / AlpC interaction *in vitro*.** (**A**) Equimolar concentrations of purified AlpA and AlpC were incubated with ATP, DNA or both ATP and DNA. Sedimentated protein was separated from non-sedimentated protein by centrifugation. The supernatant (S) and pellet (P) fractions were analysed by SDS-PAGE and visualized by coomassie staining. In the absence of ATP, AlpC does not polymerize and remains in the supernatant fraction, similar to AlpA. In the presence of ATP or ATP and DNA, AlpC polymerises and is found in the pellet fraction. Under these conditions, AlpA interacts and co-sediments with AlpC. (**B**) In the absence of AlpC, AlpA does not sediment. (**C**) Sedimentation of AlpC is dependent of nucleotide. (**D-E**) As a control, BSA was incubated with AlpC. Unlike AlpA, BSA does not co-sediment with AlpC.


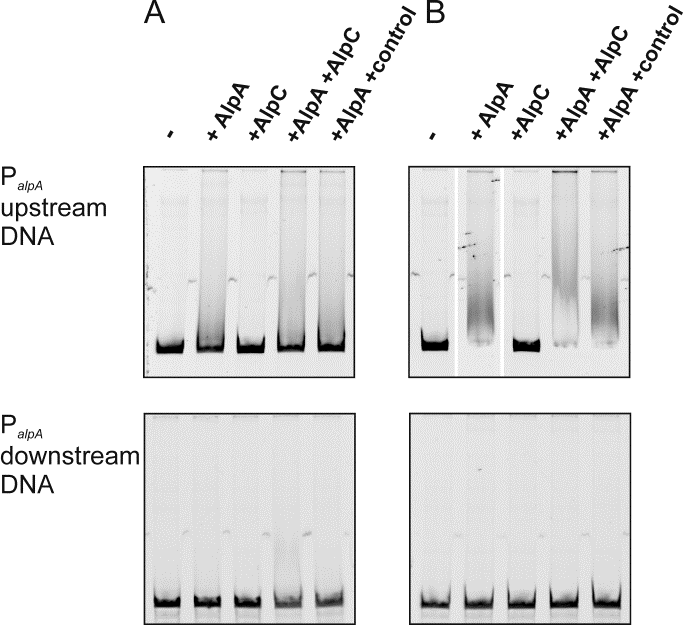


**Figure S5: Evidence for an interaction of AlpA and AlpC in the presence of the upstream region of *alpAC*.** 100 ng DNA of the upstream region of *alpAC* (500bp) containing the binding site of AlpA, *alpS,* were incubated with AlpA (200-fold molar excess) as indicated without ATP (A) or with ATP (B). AlpC (30-fold molar excess) or the control protein HrrA (2-fold molar excess) were added as indicated. (B) To induce polymerization of AlpC filaments, ATP was added to the reaction mixture. The downstream region of the *alpAC* operon (500 bp) was used as control fragment in both experiments. After20 min of incubation the samples were separated on a 10% non-denaturing polyacrylamide gel and stained with SYBR green I. No difference was observed without ATP, but the addition of ATP resulted in a super shift of AlpA-bound DNA. This provides additional evidence for a direct interaction of AlpA with AlpC filaments in the presence of *alpS* DNA.

**Figure S6**: Induction of CGP3 phage is reduced in the absence of AlpC, *in vivo*. The number of CGP3 phage DNA particles was measured in both WT (strain ATCC 13032::pLAU44-CGP3-Spec )and Δ*alpC* (strain CDC024) cells (n≥210). Cells were treated with mitomycin C to induce the CGP3 phage. On average WT cells contained 2.9 CGP3 foci per cell, while Δ*alpC* contained 1.9. ***T-test assessment shows that there is a significant difference between the mean values (p value <0.0001).

**Legend to supplemental movies**


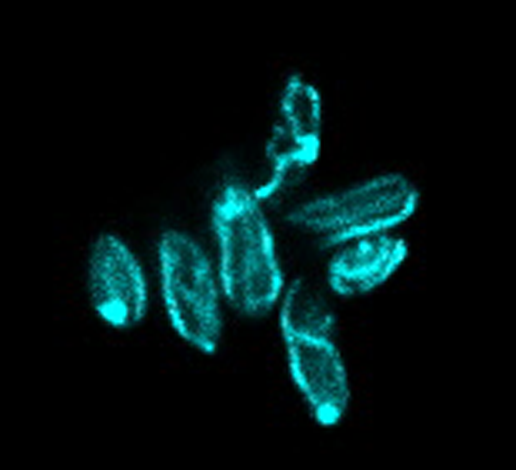


**Movie S1: AlpC assembles into filaments.** Z-stack through *C. glutamicum* cells expressing AlpC-CFP. Cells were grown to logarithmic growth phase and imaged using a Zeiss Axioimager M1 as described in material and methods. A still image is shown above.


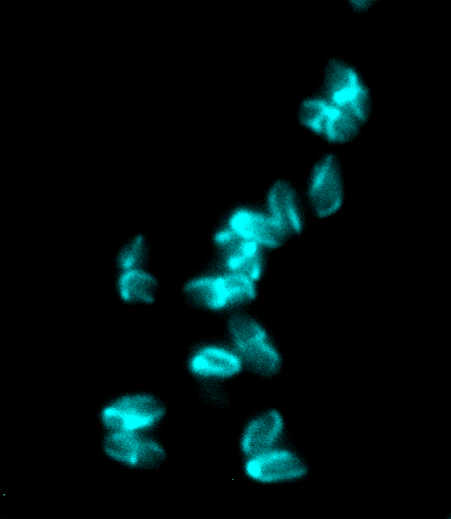


**Movie S2: AlpC filaments are dynamic.** Time lapse image series of *C. glutamicum* cells expressing AlpC-CFP. Cells were grown to logarithmic growth phase and imaged using a Zeiss Axioimager M1 as described in material and methods. A still image is shown above.


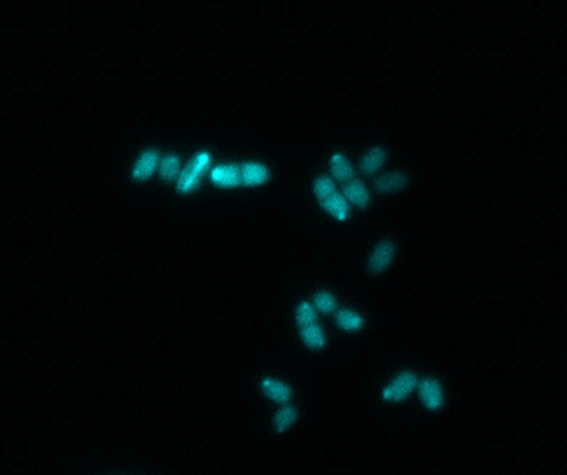


**Movie S3: AlpC filament dynamics require nucleotide hydrolysis.** Time lapse image series of *C. glutamicum* cells expressing AlpC^D301A^-CFP. Cells were grown to logarithmic growth phase and imaged using a Zeiss Axioimager M1 as described in material and methods. Many cells only show foci or patches of AlpC-CFP, but no filaments. Indeed, only 1.6% of all cells expressing AlpC^D301A^-CFP show clear filaments. Time lapse analysis shows that these filaments are not as dynamic as wild type AlpC-CFP filaments. A still image is shown above.

**Movie S4: Analysis of AlpC-mCherry filament dynamics by FRAP.** Part of AlpC-mCherry filaments were bleached using a 561 nm laser. The recovery of AlpC-mCherry in the bleached area was monitored by time lapse analysis, where images were acquired every 10 seconds prior to bleaching.

**Movie S5:** C**ontrol for dark-state-reversal of mCherry fluorophore.** AlpC-mCherry expressing cells were fixing with 1% formaldehyde for 30 minutes at room temperature prior to analysis. FRAP analysis was carried out and evaluated as described in materials and methods. A region within the cell was bleached with the 561 nm laser and recovery of AlpC-mCherry was monitored over time, with image acquisition every 10 seconds. There was no recovery of mCherry in the bleached region.

**Movie S6: Time lapse analysis of AlpA-eYFP and AlpC-mCherry**. Under CGP3 phage induction conditions, the dynamics of AlpA-eYFP and AlpC-mCherry was analyzed. Images were acquired every 5 seconds for a total of 65 seconds. The AlpA-eYFP focus moves from a centrally located position to the cell pole.

**References**

1. Niebisch, A. and Bott, M. (2001) Molecular analysis of the cytochrome bc1-aa3 branch of the *Corynebacterium glutamicum* respiratory chain containing an unusual diheme cytochrome c1. *Archives of microbiology*, **175**, 282-294.

2. Gibson, D.G., Benders, G.A., Andrews-Pfannkoch, C., Denisova, E.A., Baden-Tillson, H., Zaveri, J., Stockwell, T.B., Brownley, A., Thomas, D.W., Algire, M.A. *et al.* (2008) Complete chemical synthesis, assembly, and cloning of a *Mycoplasma genitalium* genome. *Science*, **319**, 1215-1220.

3. Frunzke, J., Bramkamp, M., Schweitzer, J.E. and Bott, M. (2008) Population Heterogeneity in *Corynebacterium glutamicum* ATCC 13032 caused by prophage CGP3. *Journal of bacteriology*, **190**, 5111-5119.

4. Kinoshita, S., Udaka, S. and Shimono, M. (1957) Studies on the amino acid fermentation: I. Production of L-glutamic acid by various microorganisms. *J. Gen. Appl. Microbiol.*, **3**, 193-205.

5. Schäfer, A., Tauch, A., Jäger, W., Kalinowski, J., Thierbach, G. and Pühler, A. (1994) Small mobilizable multi-purpose cloning vectors derived from the *Escherichia coli* plasmids pK18 and pK19: selection of defined deletions in the chromosome of *Corynebacterium glutamicum*. *Gene*, **145**, 69-73.

6. Eikmanns, B.J., Kleinertz, E., Liebl, W. and Sahm, H. (1991) A family of *Corynebacterium glutamicum*/*Escherichia coli* shuttle vectors for cloning, controlled gene expression, and promoter probing. *Gene*, **102**, 93-98.

7. Bussmann, M., Baumgart, M. and Bott, M. (2010) RosR (Cg1324), a hydrogen peroxide-sensitive MarR-type transcriptional regulator of *Corynebacterium glutamicum*. *The Journal of biological chemistry*, **285**, 29305-29318.

8. Cremer, J., Eggeling, L. and Sahm, H. (1990) Cloning the *dapA* *dapB* cluster of the lysine-secreting bacterium *Corynebacterium glutamicum*. *Mol. Gen. Genet.*, **220**, 478-480.

9. Kirchner, O. and Tauch, A. (2002) Tools for genetic engineering in the amino acid-producing bacterium *Corynebacterium glutamicum*. *Journal of biotechnology*, **104**, 287-299.
